# A randomised feasibility study of computerised cognitive training as a therapeutic intervention for people with Huntington’s disease (CogTrainHD)

**DOI:** 10.1186/s40814-020-00623-z

**Published:** 2020-06-19

**Authors:** Emma Yhnell, Hannah Furby, Rachel S. Lowe, Lucy C. Brookes-Howell, Cheney J. G. Drew, Rebecca Playle, Gareth Watson, Claudia Metzler-Baddeley, Anne E. Rosser, Monica E. Busse

**Affiliations:** 1 Neuroscience and Mental Health Research Institute, Cardiff University (NMHRI), 3rd Floor, Hadyn Ellis Building, Maindy Road, Cardiff, CF24 4HQ UK; 2 Cardiff University School of Biosciences, The Sir Martin Evans Building, Museum Avenue, Cardiff, CF10 3AX UK; 3 School of Psychology, Cardiff University Brain Research Imaging Centre (CUBRIC), Maindy Road, Cardiff University, Cardiff, CF24 4HQ UK; 4 Centre for Trials Research (CTR), Cardiff University, Neuadd Meironnydd, Heath Park, Cardiff, CF14 4YS UK; 5 Cardiff University Brain Repair Group, Life Sciences Building, Museum Avenue, Cardiff, CF10 3AX UK

**Keywords:** Huntington’s disease, Cognition, Executive function training, Cognitive training, Brain training, Feasibility, Adherence

## Abstract

**Background:**

Huntington’s disease (HD) is associated with a range of cognitive deficits including problems with executive function. In the absence of a disease modifying treatment, cognitive training has been proposed as a means of slowing cognitive decline; however, the impact of cognitive training in HD patient populations remains unclear. The CogTrainHD study assessed the feasibility and acceptability of home-based computerised executive function training, for people impacted by HD.

**Methods:**

Thirty HD gene carriers were recruited and randomised to either executive function training or non-intervention control groups. Participants allocated to the intervention group were asked to complete executive function training three times a week for 30 min for 12 weeks in their own homes. Semi-structured interviews were conducted with participants and friends, family or carers, to determine their views on the study.

**Results:**

26 out of 30 participants completed the baseline assessments and were subsequently randomised: 13 to the control group and 13 to the intervention group. 23 of the 30 participants were retained until study completion: 10/13 in the intervention group and 13/13 in the control group. 4/10 participants fully adhered to the executive function training. All participants in the control group 13/13 completed the study as intended. Interview data suggested several key facilitators including participant determination, motivation, incorporation of the intervention into routine and support from friends and family members. Practical limitations, including lack of time, difficulty and frustration in completing the intervention, were identified as barriers to study completion.

**Conclusions:**

The CogTrainHD feasibility study provides important evidence regarding the feasibility and acceptability of a home-based cognitive training intervention for people with HD. Variable adherence to the cognitive training implies that the intervention is not feasible to all participants in its current form. The study has highlighted important aspects in relation to both the study and intervention design that require consideration, and these include the design of games in the executive function training software, logistical considerations such as lack of time, the limited time participants had to complete the intervention and the number of study visits required. Further studies are necessary before computerised executive function training can be recommended routinely for people with HD.

**Trial registration:**

ClinicalTrials.gov, Registry number NCT02990676.

## Key messages regarding feasibility

- The CogTrainHD study provides important proof of principle evidence regarding the feasibility and acceptability of home-based computerised cognitive training for people impacted by HD.
- The computerised cognitive training intervention is not feasible to all participants in its current form due to variable participant adherence to the intervention.
- Important aspects to consider in future studies will be strategies to overcome variable participant adherence, participant reported lack of time and the incorporation of computerised cognitive training into routine.

## Background

Huntington’s disease (HD) is a hereditary neurodegenerative disease which is characterised by a range of symptoms including motor, psychiatric and cognitive disturbances [1, 2]. Specific cognitive disturbances, including problems with executive function, have been documented from early on in the HD disease process [3, 4]. Cognitive disturbances can be particularly troubling for both people living with HD as well as friends, family members and carers [5, 6]. Therefore, in the current absence of a disease modifying pharmacological treatment for HD, or to complement potential pharmacological interventions which may be developed in the future, computerised cognitive training interventions could help to address these symptoms and improve quality of life for HD families.

Computerised brain training in clinical populations has demonstrated mixed results [7, 8]. Findings in healthy adults have shown consistent improvement in tasks upon which they have been trained [9]; however, transfer effects into untrained tasks or functional domains have either not been studied or found to be limited [10–13]. Computerised brain training has been studied in other neurodegenerative conditions. For example, accumulating evidence suggests that this approach is feasible in people with Alzheimer’s disease (AD) [14–16], and a meta-analysis of cognitive training in AD [17] highlighted improvements in learning, memory, executive function, depression, activities of daily living and self-rated functioning. Evidence in AD further supports the view that cognitive training can be useful in improving the specific cognitive domain that is being trained [15]. Computerised cognitive training has also been extensively explored in Parkinson’s disease (PD), a systematic review and meta-analysis presented by Leung et al. [18], found this intervention to be feasible and safe in this patient group. Cognitive training has been found to improve executive function and mobility in patients with PD [19–21]. Combined motor and cognitive training interventions have also resulted in improvements in daily living for PD patients [22–24]. Key limitations in the published literature in relation to the evidence of computerised cognitive training in all patient groups include small samples sizes, the combined use of cognitive training strategies and little evidence of long-term effects or follow-up which limit the interpretation of the results.

In comparison to other more common neurodegenerative conditions such as AD and PD, there has been limited investigation of cognitive training in HD, and the potential impact of computerised cognitive training in HD remains unclear. Studies in mouse models of HD, which replicate the genetic cause of the disease and share strong phenotypic similarities with the human condition of HD, have indicated that cognitive training has the potential to modify both cognitive and motor symptoms of HD [25–27]. Studies in HD clinical populations have suggested that neurofeedback (where by the participant can see a visual representation of their neural activity and is required to respond to a task in real time) may have some benefit in comparison to activity based interventions [28]. A single arm study (with no comparator group) which specifically considered computerised working memory training in HD [29] reported beneficial effects in relation to improving working memory, although this study did not include a randomisation procedure or control group. Indeed, to date, there have been no reports of randomised studies of computerised cognitive training interventions of executive function in HD, which are important to consider the implications which having a control group may have on participant recruitment, adherence and retention.

The CogTrainHD study aimed to assess the feasibility and acceptability of computerised executive function training in HD with the inclusion of a randomisation procedure and control group. The results of the study provide key information regarding participant eligibility, recruitment, willingness to be randomised into either the intervention or control arm, retention of participants, data completeness and the acceptability of the computerised cognitive training intervention. The CogTrainHD study aimed to highlight important aspects of both study and intervention design which should be considered before progressing to a full-scale randomised study which is appropriately powered to determine the efficacy of computerised executive function training in HD.

## Methods

### Aim, design and study setting

The primary aim of the study was to determine whether computerised executive function training is feasible and acceptable for people with HD in a randomised controlled study. HD gene positive participants were recruited from the Cardiff Huntington’s Disease Research and Management Clinic at Cardiff University. Participants were recruited from February 2017 to December 2018, and a study letter and information sheet was sent to eligible participants before discussing the study documentation during their appointment. In addition, we sought to approach participants’ family members, friends and carers to complete a semi-structured interview in relation to the study to determine their views on computerised cognitive training. However, involvement of a family member, friend or carer was not a requirement for participation in the study. The study design was a randomised feasibility study of a computerised executive function training for people with HD, to be completed in the home. Participants were required to attend clinical appointments to undertake the consent, baseline and outcome assessments. All participants received additional home visits which included a battery of cognitive tests, designed to assess executive function, and participants were set up on the executive function training software, for those participants allocated the intervention group. Magnetic resonance imaging (MRI) was acquired for a subset of research participants to determine if including MRI was feasible in this study population, and the results of this will be reported separately in order to study the potential mechanism underlying the intervention. The full study protocol has previously been published and is available online [30].

### Participant characteristics

Participants were recruited into the study according to the inclusion and exclusion criteria previously described in the published protocol [30]. All participants were over 18 years of age, were able to provide informed consent, had a confirmed genetic diagnosis of HD, were participating in the ENROLL-HD study, were not currently regularly completing any computerised cognitive training, were not currently actively involved in any other interventional trial and had no other significant neurological conditions.

### The executive function training intervention

The 12-week executive function training intervention was provided by HappyNeuronPro at no cost for the purposes of the study [30]. Twelve weeks was chosen as the intervention duration based on the recommendations of the cognitive training provider. A suitable computerized device (laptop or computer) and WiFi connection were required to complete the intervention: both were available to participants on a loan basis if required. The computerised intervention was designed to be completed remotely in participants’ homes, supported by email and telephone reminders which were used as prompts for participants to undertake the executive function training.

The intervention comprised of six different tasks which were specifically aimed at training executive function, with an emphasis on working memory, planning and cognitive flexibility as previously described [30]. Participants were asked to complete executive function training for a minimum of 30 min 3 sessions a week for 12 weeks. Participants allocated to the control group were asked to continue as normal for the 12-week duration, and they also received home visits, which included conversation with the researcher and the same battery of cognitive tests as the intervention group, to control for increased social interaction.

### Study assessments

The assessments which were chosen for the study were predominantly pen and paper-based, although we sought to use a quantitative timed up and go task which was a quantitative (sensor-based and electronically controlled) version of the manual timed up and go test. The outcome measures selected for inclusion in this study (as previously described [30]) were chosen to include tasks which tested both functional ability in relation to HD, the functional domain being trained (i.e. executive function) and any transfer effects of these tasks to other cognitive (categorical verbal fluency, stroop test, trail making test, letter verbal fluency test, digit span, Tower of Hanoi, card sorting and symbol digit modality) and motor domains (timed up and go, quantitative timed up and go and the clinch token transfer test). Although this feasibility study was not designed to determine efficacy, the inclusion of a range of outcome measures was designed to inform future trials. Overall study completion was defined as participants completing all study visits, ending with the outcome assessments.

### Randomisation

Randomisation was performed using a minimisation procedure which used the Minim computer software [31] to ensure balance between the groups for categorical variables of age and cognitive function (determined by the categorical verbal fluency test). Both age and cognitive function were given equal weighting during randomisation. The minimisation and allocation procedures were performed by an independent statistician (RP) to minimise potential sources of bias.

### Statistical analysis

Demographic data are reported for all participants as well as separately for the control and intervention groups. Descriptive data includes an evaluation of eligibility, recruitment, retention rates and adherence to the intervention. As this study was an initial proof of principle feasibility study, no formal sample size calculations were completed, and blinding was not applied as the researcher leading the study also completed all of the study assessments. Participant withdrawals and loss to follow-up are also reported. The progression criteria as previously defined in the study protocol [30] detailed that successful adherence to the invention was defined as completing 12 weeks of the computerised cognitive training for a minimum of three, 30-min sessions per week, based on the manufacturers’ recommendations. Pre-defined retention rates stated that retention of over 75% of participants in the study, we would consider the study feasible, equates to 21/30 participants in the study.

### Qualitative data analysis

Semi-structured interviews from all research participants, friends, family members or carers were transcribed verbatim, anonymised and analysed using the NVivo software (NVivo qualitative data analysis software; QSR International Pty Ltd. Version 12, 2018) [32] and subsequent thematic analysis. NVivo qualitative coding software was used to manage the data.

The coding framework was discussed at length with an experienced qualitative researcher to help arrange and describe themes and sub-themes. A proportion of transcripts was double coded by the qualitative researcher and any discrepancies, where data was coded under a different theme, and were discussed at length. This allowed the coding framework to be refined. As Barbour (2001:1116) explains, the most valuable aspect of multiple coding is “the content of disagreements and the insights that discussion can provide for refining coding frames. The greatest potential of multiple coding lies in its capacity to furnish alternative interpretations and thereby to act as the “devil's advocate” ”[32]. The transcripts which were double coded and discussed were selected to include data from participants who both had and had not received the brain train games, across different timepoints (baseline and outcome assessments) and with and without associated friends, family or carers.

Ten themes were identified from participant interviews and interviews from friends, family members and carers (Table 1). The barriers, benefits and facilitators for brain training (themes 1–3) were considered the most relevant themes to impact feasibility and are therefore discussed in this paper.

**Table 1:** Qualitative research themes identified in semi-structured interviews

Themes identified in participant and friends, family and carer interviews at baseline and outcome interviews	
1. Barriers to participating in brain training.	
2. Benefits of participating in brain training.	
3. Facilitators for brain training.	
4. Ways to improve brain training/intervention.	
5. Knowledge about brain training generally.	
6. Involvement of others in brain training.	
7. Technology and brain training.	
8. Impact of brain training on the participant.	
9. Views on CogTrainHD study processes.	
10. Benefits of taking part in research in general.	

Ten overarching themes were identified from semi-structured interviews conducted during the study, and themes 1–3 are discussed in this paper

## Results

**Fig. 1:**
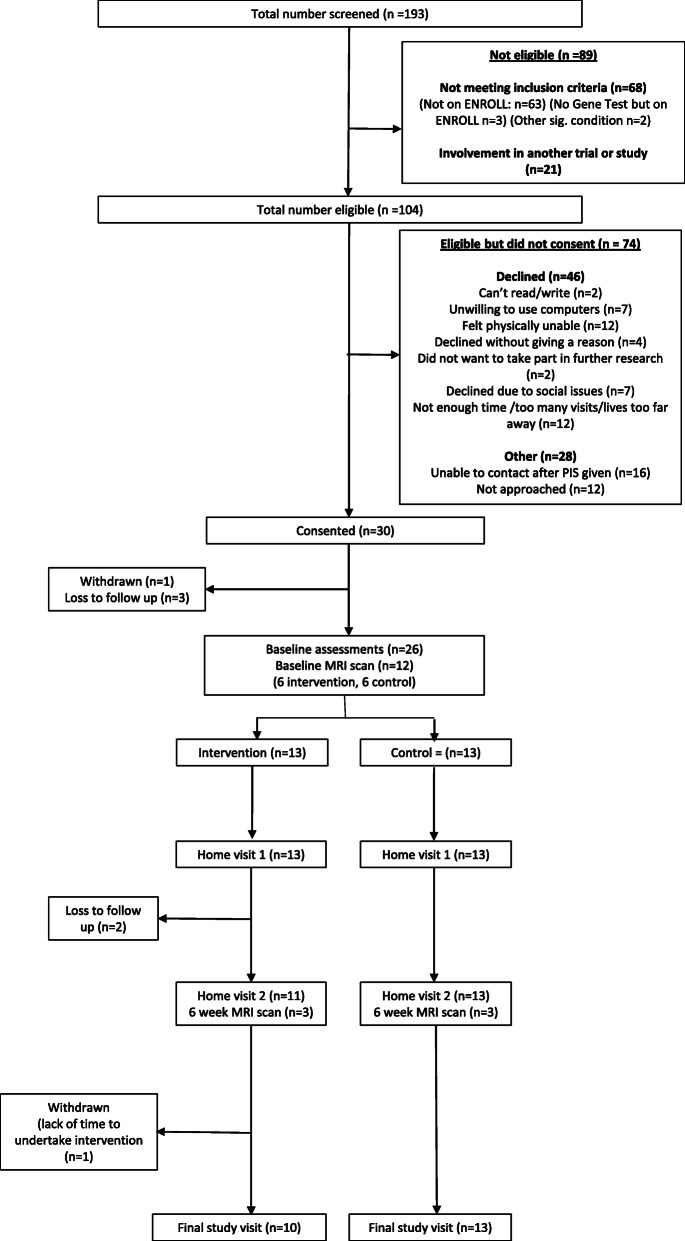
CONSORT diagram illustrating participant flow throughout the CogTrainHD study

The results of this feasibility study have been reported according to the CONSORT Extension to Pilot and Feasibility Trials [33]. The CONSORT diagram shown in Fig. 1 details the flow of participants throughout the study.

### Eligibility

A total of 193 potential participants were screened to take part in the study and 89/193 of participants screened did not meet the eligibility criteria for taking part in the study. The main reason for not meeting eligibility criteria was that potential participants were not enrolled on the ENROLL-HD study. Involvement in another trial or study, other significant neurological conditions or lack of a positive genetic test for HD was other reasons for participants not being eligible to take part in the CogTrainHD study.

### Recruitment

A total of 104 potentially eligible participants were approached to take part in the study; of those, 46 declined to take part in the study, due to reasons including not feeling that they were physically able to take part in the intervention, reasons related to lack of time, being unwilling to use computers and social issues (see CONSORT diagram in Fig. 1).

Thirty participants were recruited to take part in the study; therefore, we were unable to meet the overall recruitment target of 50 participants. Twenty-six participants completed the baseline study assessments, 3 were lost to follow-up (without explanation) and 1 withdrew due to health reasons which were unrelated to HD, prior to completing baseline assessments. Of the remaining 26 participants, 13 were randomised to the control group, and 13 were randomised to the intervention group. In the intervention group, 2 participants were lost to follow-up after completing the first home visit (without explanation), and 1 withdrew after the second home visit due to lack of time. Ten out of 13 participants allocated to the intervention group completed the study. All participants who were allocated to the control group (13/13) completed the study (Fig. 1 and Table 4).

### Participant demographics

Participant characteristics of age, gender, handedness and the number of years spent in education were similar between the control and intervention groups and reflective of the demographics anticipated for the HD patient population (Table 2). Participant characteristics of CAG repeat length were similar between both groups. Symbol digit modality scores which are indicative of cognitive function at baseline were higher in the control group compared to intervention, although the standard deviation of the measures was high. Total functional capacity which is indicative of disease severity demonstrated that overall disease severity was slightly worse in the intervention group; although given the small sample size, there are considerable variations within the data.

**Table 2:** Table of demographic data for study participants

Demographic characteristics	Overall	Control group	Intervention group
	(*n* = 26)	(*n* = 13)	(*n* = 13)
Age (y), mean (SD)	45.8 (11.1)	45.6 (11.6)	45.9 (11.2)
Gender (male/female, *n*), (%)	13/13, (54)	7/6, (54)	6/7, (46)
Alcohol intake (units per week), mean (SD)	5.7 (6.7)	6.9 (8.0)	4.6 (5.0)
Smoking (cigarettes per day), mean (SD)	2.0 (5.1)	0.7 (2.8)	3.3 (6.6)
Caffeine (yes/ no, *n*), (%)	23/26 (88%)	13/0 (100%)	10/3 (77%)
Handedess (R/ L, *n*) (%)	22/4, (85%)	11/2, (85%)	11/2, (85%)
Years in Education (y), mean (SD)	14.9 (4.9)	14.4 (5.4)	15.4 (4.6)
CAG Repeat Length	43.2 (2.4)	43.1 (2.2)	43.3 (2.7)
Symbol Digit Modality (SDMT) Score^a^, mean (SD)	39.8 (16.2)	44.4 (17.3)	35.5 (14.5)
Total Functional Capacity (TFC) Score^a^, mean (SD)	11.0 (2.9)	12.0 (1.8)	10.2 (3.4)
**Outcome Clinical Characteristics**			
Categorical verbal fluency score, mean (SD)	18.1 (6.2)	19.4 (6.9)	16.8 (5.3)
HDProTriad Total Score^a^, mean (SD)	5.5 (1.4)	4.6 (0.9)	6.3 (1.4)
HDPro Triad Total Motor Score^b^, mean (SD)	1.4 (0.6)	1.1 (0.2)	1.7 (0.7)
HDPro Triad Total Cognitive Score^b^, mean (SD)	2.1 (0.8)	1.7 (0.5)	2.4 (0.8)
HDPro Triad Emotional Score^b^, mean (SD)	2.0 (0.6)	1.7 (0.5)	2.3 (0.6)

^a^Participant data for CAG repeat length, symbol digit modality test and total functional capacity score are taken from the most recent ENROLL-HD visit of the participant

^b^The HDProTriad questionnaire was developed for use in HD and measures motor function, cognition and emotion. Higher scores indicate a greater degree of impairment, and a score of one indicates normal function

Clinical characteristics of the participants demonstrated a higher average categorical verbal fluency score in the control group in comparison to the intervention group. Self-reported scores of motor function, cognition and emotion measured in the HDProTriad questionnaire [34] showed a greater degree of impairment in the intervention group in comparison to the control group. However, it is important to note the relatively small sample size with associated variability between individual participants, which is reflected in the high degree of standard deviation within the data (Table 2).

### Study assessment completion

Participant completion rates for motor assessments, questionnaires and cognitive assessments throughout the study are shown in Table 3. The timed up and go and quantitative timed up and go assessments could only be completed by participants who were able to walk: one participant used a wheelchair and was unable to complete the assessments which required walking. Furthermore, technical issues with the quantitative timed up and go apparatus including error messages resulted in lower completion rates for quantitative timed up and go than that of the timed up and go. The clinch token transfer test had a 100% completion rate between both study groups. Study questionnaires were completed to a high degree (Table 3), although there were some instances of participants missing questions. The majority of participants with incomplete data declined to complete the questionnaires when asked, typically due to time constraints or an unwillingness to answer questions relating to mood. The majority of cognitive assessments were completed in their entirety (Table 3), although some participants declined to complete the categorical verbal fluency tasks as they found this task particularly frustrating. Semi-structured interviews were completed by the majority of participants, although rates of friend and family member of carer completion were significantly lower than those of participants, as many participants attended their research visits without friends, family members or carers.

**Table 3:** Table of assessment completion for study participants

	Consent visit	Baseline visit	Home visit 1	Home visit 2	Outcome visit
	(*n* = 30)	(*n* = 26)	(*n* = 26)	(*n* = 24)	(*n* = 23)
**Motor assessments**					
Timed up and go		24/26			22/23
Quantitative timed up and go		17/26			19/23
Clinch token transfer test		26/26			23/23
Questionnaires					
International Physical Activity Questionnaire		26/26			23/23
Life Space Assessment		26/26			96%
HDProTriad		26/26			91%
Hospital Anxiety Depression Scale		26/26			96%
Sociodemographic Questionnaire		26/26			100%
**Cognitive assessments**					
Categorical verbal fluency test		26/26	26/26	24/24	23/23
Stroop test		26/26	26/26	24/24	23/23
TrailMaking A Test		26/26	26/26	24/24	23/23
TrailMaking B test		26/26	26/26	24/24	23/23
Letter verbal fluency test (F)		26/26	24/26	23/24	22/23
Letter verbal fluency test (A)		26/26	24/26	23/24	22/23
Letter verbal fluency test (S)		26/26	24/26	23/24	22/23
Symbol digit modality test		26/26	26/26	24/24	23/23
Digit span			26/26	23/24	23/23
Single Box Crossing Task			26/26	24/24	23/23
Dual Box Crossing Task			26/26	24/24	23/23
Tower of Hanoi			26/26	24/24	23/23
Card Sorting Test			26/26	24/24	23/23
**Semi-structured interviews**					
Participant semi-structured interview	29/30				22/23
Friend or Family Member semi-structured interview	12/30				9/23

### Descriptive statistics for baseline, home visit and outcome measures

Summary statistics for all baseline, home visit and outcome measures completed during the study are shown in Additional file 1. Interpretation of these data was not within the remit of this small scale feasibility study, given the small sample size and high degree of variability in the data due to the range of disease stages included in the feasibility study and the range of participant adherence shown to the executive function training intervention.

### Retention of study participants

Retention of study participants decreased during the study (Table 4), a notable loss to follow-up was observed after the consent and subsequent baseline visit (also see CONSORT diagram in Fig. 1). Following randomisation, all participants in the control group were retained for the duration of the study; however, the intervention group had 2 participants whom were lost to follow-up, and 1 participant, who withdrew, stating that they did not have time to complete the executive function training.

**Table 4:** Study retention

Time (w)	Study Retention overall, *n*/*n*	Retention intervention, *n*/*n*	Retention control, *n*/*n*
Consent	30/30		
0	26/30		
6	24/30	11/13	13/13
12	23/30	10/13	13/13

The study protocol stated that the intervention would be considered feasible if overall retention was greater than 75% (Table 4). Although a retention rate of greater than 75% was attained, this finding must be considered in light of the variable participant adherence to the intervention (Table 5). No harms or serious adverse events (SAEs) were reported for research participants involved in the study.

**Table 5:** Participant adherence to the intervention over 12 weeks

	Weeks of intervention (time, mins)												
Participant Code	1	2	3	4	5	6	7	8	9	10	11	12	Average time per week (mins)
**005**	0	0	0	0	0	0	0	0	0	0	0	0	Loss to follow up
**006**	90	60	90	60	0	180	120	60	60	180	90	60	87.50
**007**	60	45	120	110	90	90	90	90	90	90	60	30	80.41
**010**	90	50	60	60	60	30	0	240	30	60	130	60	72.50
**011**	0	30	2	0	0	0	36	40	0	0	0	0	9.00
**013**	15	120	80	90	32	Withdrawn							Withdrawn
**016**	60	0	0	0	0	0	0	0	0	0	0	0	Loss to follow up
**017**	3	0	0	0	0	0	0	0	0	0	0	0	0.25
**020**	103	104	101	97	96	117	51	85	106	123	111	108	100.20
**023**	128	138	110	279	115	123	143	80	117	107	90	0	119.20
**027**	152	243	55	85	49	58	88	130	122	95	102	164	111.92
**029**	46	29	59	63	31	35	62	70	36	24	31	30	43.00
**030**	33	88	102	94	133	89	233	110	108	94	106	123	109.42

### Participant adherence to executive function training

All participants in the intervention group were asked to complete three 30-min sessions of executive function training each week. Participant adherence to the training was measured across the 12-week intervention duration. The average time per week spent playing the executive function training games averaged across participants that completed the study was 73.3 min per week with a range of 0.25–119.2 min (Table 5). Participants were offered the choice of email or telephone alerts to remind them to complete the intervention, and all participants chose to receive email reminders.

Four out of 10 participants in the intervention group successfully adhered to the executive function training intervention for an average of 90 min per week or more, which was less than the one hundred percent specified in the original progression criteria stated to determine feasibility. Therefore, based on the variability of participant adherence, the computerised executive function training intervention cannot be considered feasible in its current form.

### Qualitative results

All participants in the study and associated friends, family members or carers were invited to complete a semi-structured interview at the beginning and end of the study. Twenty-nine out of 30 participants completed the baseline semi-structured interview, and 12/30 participants had a friend, family member or carer that consented to completing a semi-structured interview at the beginning of the study. Upon completion of the study 22/23 participants completed the semi-structured interview at the end of the study, one participant in the intervention group declined to take part in the interview due to lack of time, and all participants in the control group completed the interview at the end of the study. Friend, family member and carer completion of semi-structured interviews at the end of the study reduced to 9/23, reasons given for declining to take part all centered around lack of time and therefore the participant attending the final study visit alone.

### Intervention benefits

Thematic analysis of the qualitative data gathered from semi-structured interviews revealed that study participants had perceptions at baseline that computerised executive function training would be beneficial to them. In the semi-structured interviews, 15/29 participants and 5/12 friends, family members or carers reported that their respective participants enjoyed taking part in the study, regardless of group assignment. Additional benefits reported in the intervention group (*n* = 10) included improved emotional well-being (*n* = 5), the positive involvement of friends and family members in the intervention (*n* = 2), that the intervention was novel (*n* = 1), self-reported improvement in the intervention performance (*n* = 5), the sense of achievement after completing the executive function training (*n* = 1), that the intervention gave participants time for themselves to destress and focus on themselves (*n* = 2) and that the participant looked forward to completing the executive function training (*n* = 1). Table 6 denotes illustrative examples of the benefits described by participants who were allocated to the executive function training intervention and completed their outcome interview.

**Table 6:** Illustrative examples of participant reported benefits of the intervention

Benefits	Participant ID	Illustrative Quotes
Emotional well-being	010	“Umm, I have enjoyed doing it since it started, although it is frustrating every now and again, but on the whole it has been, a real buzz and a real bonus.”
	023	“I thoroughly enjoyed it I was enthused and it made me feel a lot better about myself and good reactions.” “Well they (*the brain training games*) allowed me to analyse, communicate to myself, talk out loud to myself, and then I had that time set three times a week and it really worked….So I guess it pepped me up.”
	030	“Now I feel kind of much more positive about it (brain training) and these kind of things now, having done them.” “I just think I kind of wanted to see what it was like actually and it was kind of more umm more you know interesting than I thought it was guna be yeh I thought it was definitely worthwhile.”
Involvement of friends/family members	027	“Yeh, I tell everybody about it and they all enjoy listening to what I have been doing and yeh it is true isn’t it, it is a help.”
Novelty	029	“at the start it was good. It was something different…”
Self-reported improvement	007	“Like myself I can see benefits from it. Like I said numeracy, I deal with numbers a lot and different things. I can see a benefit that way.” “I just liked doing the different training and keeping my brain active.” “I just liked using my brain a bit more than it actually does.”
Sense of achievement	020	“it was nice when it all came together you know, it was fun, you’d achieved something really.”
Time for participant	006	“I just sat quietly and did the training, so it’s been fine.” “And that was it and I also appreciated that it was it was nice you know what I mean. I was sort of left alone as well to do your own thing. Although, sounds a bit harsh, but you know it is nice to have peace and quiet, it is something that you have got to do.”

Reported benefits are grouped according to theme and presented in alphabetical order

### Intervention barriers

Thematic analysis of the qualitative data at the beginning of the study revealed that participants perceived barriers to completing the executive function training. At baseline, some participants felt that computerised executive function training games may be scary (*n* = 1) or addictive (*n* = 5); however, only one participant described the computer games as potentially addictive in their outcome interview (*n* = 1). The most widely reported participant barrier to completing executive function training at baseline was lack of time (*n* = 12), typically due to work commitments or caring responsibilities for dependents. Other barriers noted by participants and friends, family members or carers included the impact of disease stage on ability to physically be able to complete the executive function training games (*n* = 9), becoming tired after completing the intervention (*n* = 2) or the potential cost of the intervention software (*n* = 1) as well as the potential to cheat the intervention (*n* = 4).

At the end of the study, participants allocated to the intervention group (*n* = 13) reported barriers which impacted on their ability to complete executive function training. Barriers relating to the executive function games themselves included that the computer games were tedious (*n* = 2), difficult or frustrating (*n* = 1), general glitches or problems using technology (*n* = 4), becoming tired after completing the intervention (*n* = 1) and lack of time to complete the intervention (*n* = 1).

Table 7 denotes illustrative examples of barriers described by participants who were allocated to the executive function training intervention who successfully adhered to the intervention and completed their outcome interview.

**Table 7:** Illustrative examples of participant reported barriers to completing the intervention.

Barriers	Participant ID	Illustrative quotes
Addictive	030	“…because it is kind of limited time and you know so I was on there … all night a couple of times. But also cos yes you it does become a bit addictive you know.”
Difficult	027	“I found that (the computer games) quite difficult to begin with because I didn’t know what to expect really”.
Lack of time	006 007 007 007 011 020 029 029	“it has just been difficult trying to fit everything in. But, other than that it is fine.” “I mean it is sort of like everything, you’ve got to fit it in and I am quite busy.” “I thoroughly enjoyed it, yeh I will be honest with you. I wish I had more time for it.” “I think it’s definitely something you need to find your own time to do it.” “Umm, I played them enough as I could…I have a busy life.” “Umm, I don’t think so, just finding the time to fit in in really.” “Finding the time to actually do it was the worst thing for me.” “It was some weeks it was pretty impossible, just managing to do one was just thing, but I suppose if you had enough time one your hands it is easy enough.”
Technology	010 023 017	“I was playing it for hours on end and it wasn’t recording.” “Unfortunately it took me a while to get used to the mouse. I used, the computer mouse.” “I couldn’t do it could I because I didn’t have no WiFi.”
Tedious	029	“It was alright. But it was a bit tedious towards the end, just the same thing was quite repetitive all the time. But yeh it was alright not too bad.”

Reported barriers are grouped according to theme and presented in alphabetical order

### Intervention facilitators

Upon completion of the study, participants allocated to the intervention recognised numerous facilitators which helped them engage with and adhere to the intervention (Table 8). Those who were most successful in adhering to the executive function training programme were able to successfully incorporate the training into their daily routine (*n* = 5), and they often had the help and support of friends, family members or carers (*n* = 2). Participants described particular attributes which facilitated their adherence to the executive function training, and these included commitment (*n* = 2 participant, *n* = 2 friends, family members or carers), competitiveness (*n* = 4 participants), motivation (*n* = 8 participants), perseverance (*n* = 2) and determination (*n* = 1 participant).

**Table 8:** Illustrative examples of participant reported facilitators in adhering to and completing the intervention

Facilitators	Participant ID	Illustrative Quotes
Commitment	010	“you have got to have that commitment to it, for that half an hour. Umm, because if you don’t then err, you’re not going to get the results from it.”
Determination	006 007	“ I just catch up when I am, I’ve not done it, but umm I haven’t worried about not doing it, I just knew that I then had to catch it up to try and keep on track.” “I love a challenge. I don’t give up on a challenge until I thoroughly complete it.”
Diagnosis	010	“And obviously if I keep on at it …. that’ll help me in my later years, you know. Umm, with the diagnosis in front of me really.”
Help/support	011 020	“Yeh, she (family member) was helping me, showing me what to do.” “(it impacted on those around me)… with everyone’s help that I needed.”
Interest in the results of the study	027	“It will be interesting to see if there are positive results in the motor skills that would be interesting wouldn’t it for other people.”
Motivation	010 023	“I think it has built up my motivation and err that err, yeh it really has motivated me in a big big way.” “It allowed me to get up out of bed and it motivated me.”
Routine	007 010 029 030	“I mean it is sort of like everything, you’ve got to fit it in and I am quite busy…it didn’t take a huge amount of effort as such, as long as you do, be flexible with your time.” “(when did you play the brain training games?) Lunchtime mostly” “I just catch up when I am, I’ve not done it, but umm I haven’t worried about not doing it, I just knew that I then had to catch it up to try and keep on track.” “I tended to kind of like do them at fairly similar sort of times you know sort of not late at night or kind of just you know errr yeh. Cos sometimes you know it would take a bit longer than expected, but you know I tried to generally sort of do it you know sort of like umm late morning lunchtime ish most days.”

Reported facilitators are grouped according to theme and presented in alphabetical order

## Discussion

The CogTrainHD study implements a randomised controlled study design in the feasibility and evaluation of computerised executive function training with people and families impacted by HD. The results suggest that computerised executive function training is not feasible in its current form, due to variable participant adherence to the intervention, although the findings demonstrated that the intervention did not cause any harm, as no SAEs were reported. Participant retention within the study was high overall, although notably, retention was comparatively lower in the intervention group (10/13) in comparison to the control group (13/13), and participant adherence to the intervention was variable (Table 5). Patients reported time limitations as the driving factor for non-compliance with the computerised cognitive training intervention. The results of the present study should be considered in light of this alongside the comparatively small sample size. The intervention was provided with at no cost for the purposes of the study, although the software is likely to incur a cost in the longer term, which is something that should be carefully considered in terms of the longer term approach to whether cognitive training may be a feasible option in this patient population. The study has generated important proof of principle insights into interventional study design which can be used to inform the delivery of future studies that consider non-pharmacological interventions in the HD patient population.

In the current absence of a pharmacological treatment for HD, the CogTrainHD study described here explored the feasibility and acceptability of computerised executive function training. The results of the study should be viewed in light of ongoing research which seeks to specifically target the mutant huntingtin protein through huntingtin lower strategies [35–39]. Computerised cognitive training is not designed to specifically target the genetic cause of HD, although there is potential value in combining such behavioural interventions with ongoing pharmacological studies.

### Study recruitment

Thirty participants were recruited to take part in the study; therefore, we were unable to meet the original sample size target of fifty participants. Eligibility criteria for the study, specifically involvement in the ENROLL-HD study, were an inclusion criterion for the study so that additional research data regarding the participants could be considered alongside the potential for long-term follow-up. However, this may need to be reconsidered given the large number of potential participants who were not eligible. Recruitment to the study should also be considered in light of the presence of other clinical studies, which potentially eligible participants were able to consent to which subsequently excluded them from subsequent involvement in the CogTrainHD study. HD is a rare disease, thought to affect ~ 12 in 100,000 people in the UK [40]; given the complexity of living with HD, recruitment to research studies can be challenging. In the future, additional sites could be included to provide a larger pool of potential participants which may increase the rate of recruitment.

### Assessment completion

Initial loss to follow-up and withdrawal of participants prior to randomisation and subsequent allocation to group (4/30) impaired the overall feasibility of the study. However, overall, the assessments that participants were asked to complete during the study were completed to a high degree of completeness accuracy. However, there were notable technical difficulties with the quantitative timed up and go apparatus, and the sensors did not connect to the recording device, which resulted in incomplete data. The apparatus provider was contacted, and these technical studies were resolved during the study. The manual timed up and go version of the task was completed to a higher degree (Table 4), although there were cases where participants were physically unable to complete the motor tests due to using a wheelchair, which is understandable given the broad range of disease stages included in the current study and the significant motor difficulties shown by some participants.

Questionnaires were self-completed by participants. There were occasions where participants did not fully complete questionnaires, and this could be resolved by introducing a researcher to complete the questionnaires with participants, however, this suggestion must be balanced with participant privacy and the subsequent accuracy of the results obtained. Furthermore, in the future, it may be relevant to complete some assessments by telephone, online or by proxy. Although, these assessment completion methods all have pros and cons, including the availability of people to act as proxy, the availability of participants, technical issues and time limitations. The categorical verbal fluency test results were comparable to normative data previously described [41]. Despite this, several participants declined to complete the categorical verbal fluency tasks as they found them frustrating, and this was particularly notable in the home visit assessments (Table 2). Summary statistics of assessments completed in clinic and at home (Additional file 1) do not indicate any differences in the results of the assessments completed at home in comparison to those completed in the clinic.

### Improving participant adherence to the executive function training intervention

A key finding of the CogTrainHD study was variable participant adherence to the intervention (Table 4). In order to remedy this, several participants suggested (in their semi-structured interviews) increasing the portability of the executive function training software, for example, by making the intervention available on tablet computers and mobile telephones. This participant suggestion would be interesting to incorporate into future studies, although it should be considered in light of the additional variables which portability may introduce including variable environments in which the training is completed, heterogeneity in device speed and physical dimension, software compatibility issues and issues regarding digital literacy of the population [42]. One of the reasons given for not wishing to take part in the present study was an unwillingness to use computers (Fig. 1). For these individuals, digital training applications may introduce an additional cognitive burden which is not present for more digitally literate patients. Therefore, future studies could also look to explore computerised training interventions in comparison to more traditional pen and paper-based approaches.

Several participants noted benefits to completing the executive function training (Table 7) that included having space and time for themselves and introducing more portable options for the intervention may prevent this. However, we were unable to interview participants who were lost to follow-up or decided to withdraw from the study; therefore, their views are not reflected in the results obtained. The benefits described by participants may have been due to the fact that they were engaged in the intervention and therefore had something novel and engaging to undertake. Furthermore, there are additional logistical issues to undertaking computerised training, such as access to roaming data if mobile devices are used in addition to the associated cost of the digital equipment and the cognitive training software. Further, studies could look to explore individual participant preferences and using digital technologies to personalise the intervention to improve participant adherence [43]. Participants who incorporated the executive function training into their routine and had the involvement of a friend, family member or career were more likely to successfully adhere to the intervention. Therefore, future studies could consider the involvement of friends, family, or carers are a possible inclusion criterion to improve participant adherence.

## Future definitive trials

The CogTrainHD study is the first randomised controlled study to consider computerised executive function training in HD in relation to a usual care comparator. Despite the small sample size included in the current study, the presence of a control group is vital to assess the willingness of participants to be randomised to different groups within the study and to explore the variability of outcomes in different groups. Furthermore, the study retention data indicate that participants understood the need for a control group, and that allocation to the control group did not negatively impact study retention; this was also reflected in the qualitative data. Further work is required to explore the inclusion of additional study groups (if sufficient numbers allow) to explore the effects of home visits, time on a computer and support provided by friends, family members and carers. Assessment completion did not differ between the study visits conducted in clinic or within the home, and this may have implications for the design of a definitive trial, particularly in targeting the often, limited resources which are available.

## Conclusions

The results of the CogTrainHD study show that computerised executive function training administered in participant homes and supported by email reminders does not cause any harms, although it is not feasible in its current form. Participant adherence to the intervention was variable, and future studies could seek to improve adherence by exploring the portability of the intervention, developing a more stimulating and engaging cognitive training intervention as well as exploring the support provided by friends, family members and carers to allow participants to engage in the study and adhere to the intervention.

## Supplementary information

**Additional file 1: Table S1.** Descriptive data for all baseline measurements. **Table S2.** Descriptive data for home visit assessments at home visit one. **Table S3.** Descriptive data of home visit assessments for home visit two. **Table S4.** Descriptive data for all outcome measurements.

## Data Availability

The datasets used and/or analysed during the current study are available from the corresponding author on reasonable request.

## Abbreviations

CONSORT: Consolidated Standards of Reporting Trials Investigator
HD: Huntington’s disease
IC: Informed Consent
REC: Research Ethics Committee
SAE: Serious adverse event
