# Supplementary material for: A randomised feasibility study of computerised cognitive training as a therapeutic intervention for people with Huntington’s disease (CogTrainHD)
Source: Pilot Feasibility Stud. 2020 Jun 19;6:88. doi: 10.1186/s40814-020-00623-z (PMC7304172; doi:10.1186/s40814-020-00623-z)
Supplement: Supplementary file 1 — Additional file 1: Table S1. Descriptive data for all baseline measurements. Table S2. Descriptive data for home visit assessments at home visit one. Table S3. Descriptive data of home visit assessments for home visit two. Table S4. Descriptive data for all outcome measurements. [file 40814_2020_623_MOESM1_ESM.docx]

**Appendix One**

**Table 1 - Descriptive data for all baseline measurements**

| Baseline Measures  (n=26) | Overall  (n=26) | Control Group  (n=13) | Intervention Group  (n=13) |
| --- | --- | --- | --- |
| **Motor Tasks** |  |  |  |
| **Timed Up and Go** |  |  |  |
| TUG_Baseline_Time To Rise, mean (SD) | 0.90 (0.4) | 0.88 (0.4) | 0.93 (0.4) |
| TUG_Baseline_Time To Walk, mean (SD) | 8.2 (1.8) | 8.0 (2.2) | 8.3 (1.4) |
| TUG_Baseline_Time To Complete, mean (SD) | 9.0 (2.1) | 8.9 (2.5) | 9.1 (1.7) |
| Alphabet_Baseline, mean (SD) | 6.9 (3.2) | 6.9 (3.3) | 7.0 (3.2) |
| TUG_Motor_Time To Rise, mean (SD) | 0.9 (0.4) | 0.9 (0.3) | 0.9 (0.5) |
| TUG_Motor_Time To Walk, mean (SD) | 8.6 (2.9) | 8.5 (3.7) | 8.7 (1.8) |
| TUG_Motor_Time To Complete, mean (SD) | 9.6 (3.3) | 9.7 (4.3) | 9.6 (1.9) |
| TUG_Cog_Time To Rise, mean (SD) | 0.9 (0.4) | 0.9 (0.3) | 0.9 (0.5) |
| TUG_Cog_Time To Walk, mean (SD) | 7.7 (2.0) | 7.0 (2.0) | 8.6 (2.0) |
| TUG_Cog_Time To Complete, mean (SD) | 9.8 (5.2) | 10.0 (6.7) | 9.6 (2.8) |
| TUG_Triple_Time To Rise, mean (SD) | 0.9 (0.4) | 1.0 (0.5) | 0.9 (0.4) |
| TUG_Triple_Time To Walk, mean (SD) | 8.8 (3.4) | 8.8 (4.1) | 8.9 (2.3) |
| TUG_Triple_Time To Complete, mean (SD) | 10.0 (3.9) | 10.0 (4.4) | 10.0 (3.3) |
| **Quantitative Timed Up and Go** |  |  |  |
| QTUG_Baseline_Time To Complete, mean (SD) | 9.7 (3.9) | 10.4 (8.9) | 8.7 (1.7) |
| QTUG_Baseline_Time To Stand, mean (SD) | 1.0 (0.3) | 0.9 (0.3) | 1.0 (0.4) |
| QTUG_Baseline_stride, mean (SD) | 1.2 (0.3) | 1.3 (0.3) | 1.1 (0.2) |
| QTUG_Baseline_velocity, mean (SD) | 135.7 (28.5) | 131.2 (35.0) | 142.2 (15.9) |
| QTUG_Baseline_Time To Turn, mean (SD) | 2.4 (0.7) | 2.5 (0.8) | 2.4 (0.6) |
| QTUG_Baseline_STT, , mean (SD) | 1.4 (0.6) | 1.2 (0.4) | 1.7 (0.8) |
| QTUG_Motor_Time to Stand, mean (SD) | 1.2 (0.8) | 1.3 (1.0) | 1.0 (0.5) |
| QTUG_Motor_Time To Complete, mean (SD) | 9.6 (3.7) | 10.3 (4.7) | 8.7 (1.1) |
| QTUG_Motor_stride, mean (SD) | 1.2 (0.2) | 1.2 (0.18) | 1.1 (0.2) |
| QTUG_Motor_velocity, mean (SD) | 132.0 (29.0) | 127.4 (33.0) | 140.0 (23.0) |
| QTUG_Motor_Time To Turn, mean (SD) | 2.4 (0.9) | 2.5 (1.0) | 2.3 (0.7) |
| QTUG_Motor_STT, mean (SD) | 1.7 (0.8) | 2.0 (0.9) | 1.3 (0.5) |
| QTUG_Cog_Time To Complete, mean (SD) | 10.1 (6.7) | 10.7 (8.4) | 9.4 (3.6) |
| QTUG_Cog_Time To Stand, mean (SD) | 0.95 (0.4) | 1.01 (0.4) | 0.9 (0.4) |
| QTUG_Cog_stride, mean (SD) | 1.2 (0.3) | 1.29 (0.3) | 1.1 (0.1) |
| QTUG_Cog_velocity, mean (SD) | 130.9 (27.9) | 129 (34.1) | 133.5 (17.6) |
| QTUG_Cog_Time to turn, mean (SD) | 2.8 (2.4) | 3.3 (3.1) | 2.1 (0.5) |
| QTUG_Cog_STT, mean (SD) | 2.0 (1.2) | 1.9 (1.3) | 2.1 (1.1) |
| QTUG_Dual_Time to complete, mean (SD) | 10.2 (4.5) | 10.3 (4.9) | 10.1 (4.3) |
| QTUG_Dual_Time to sit, mean (SD) | 1.1 (0.4) | 1.2 (0.4) | 1.0 (0.4) |
| QTUG_Dual_stride, mean (SD) | 1.3 (0.4) | 1.3 (0.5) | 1.2 (0.2) |
| QTUG_Dual_velocity, mean (SD) | 124.1 (32.4) | 122.3 (37.2) | 126.6 (26.8) |
| QTUG_Dual_Time to turn, mean (SD) | 2.8 (1.3) | 2.7 (1.5) | 2.9 (1.2) |
| QTUG_Dual_STT, mean (SD) | 1.9 (1.0) | 1.6 (1.0) | 2.3 (1.0) |
| **Clinch Token Transfer Test (C3T)** |  |  |  |
| C3T_Gender (male; female), n (%) | 13:13 (50) | 7:6 (54) | 6:7 (46) |
| C3T_Handedness (R; L), n (%) | 22:4, (85) | 11:2, (85) | 11:2, (85) |
| C3T_Baseline_TimeTaken, mean (SD) | 29.4 (56.7) | 18.0 (6.5) | 40.7 (79.9) |
| C3T_Baseline_RuleError, mean (SD) | 0 (0) | 0 (0) | 0 (0) |
| C3T_Baseline_DroppedTokens, mean (SD) | 0 (0) | 0 (0) | 0 (0) |
| C3T_Baseline_TransferError, mean (SD) | 0 (0) | 0 (0) | 0 (0) |
| C3T_TokenValue_Baseline_TimeTaken, mean (SD) | 7.5 (4.8) | 7.1 (4.3) | 8.0 (5.5) |
| C3T_TokenValue_Baseline_Attempted, (yes; no), n (%) | 26:0 (100) | 13:0 (100) | 13:0 (100) |
| C3T_TokenValue_Baseline_PassFail, (pass; fail), n (%) | 25:1 (96) | 13:0 (100) | 12:1 (92) |
| C3T_Alphabet_Baseline_TimeTaken, mean (SD) | 7.2 (3.8) | 6.5 (2.7) | 7.9 (4.7) |
| C3T_Alphabet_Baseline_CorrectAnswers, mean (SD) | 26 (0) | 26 (0) | 26 (0) |
| C3T_Alphabet_Baseline_Error, mean (SD) | 0 (0) | 0 (0) | 0 (0) |
| C3T_Alphabet_Baseline_Attempted, (yes; no), n (%) | 25:1 (96) | 13:0 (100) | 12:1 (92) |
| C3T_Alphabet_Baseline_PassFail, (pass; fail), n (%) | 25:1 (96) | 13:0 (100) | 12:1 (92) |
| C3T_DualTask_TimeTaken, mean (SD) | 20.6 (9.4) | 18.6 (6.8) | 22.8 (11.5) |
| C3T_DualTask_RuleError, mean (SD) | 0 (0) | 0 (0) | 0 (0) |
| C3T_DualTask_DroppedTokens, mean (SD) | 0 (0) | 0 (0) | 0 (0) |
| C3T_DualTask_TransferError, mean (SD) | 0 (0.2) | 0 (0) | 0.1 (0.3) |
| C3T_DualTask_Attempted, (yes; no), n (%) | 25:1 (96) | 13:0 (100) | 12:1(92) |
| C3T_DualTask_PassFail, (pass; fail), n (%) | 25:0 (100) | 13:0 (100) | 12:0 (100) |
| C3T_TripleTask_TimeTaken, mean (SD) | 21.3 (12.1) | 19.2 (7.2) | 23.6 (15.9) |
| C3T_TripleTask_AlphabetCorrectAnswers, mean (SD) | 25.9 (0.4) | 25.8 (0.6) | 26 (0) |
| C3T_TripleTask_Rule_Error, mean (SD) | 0 (0.2) | 0 (0) | 0.1 (0.3) |
| C3T_TripleTask_AlphabetErrors, mean (SD) | 0.1 (0.4) | 0.2 (0.6) | 0 (0) |
| C3T_TripleTask_TransferError, mean (SD) | 0.1 (0.3) | 0.1 (0.3) | 0.1 (0.3) |
| C3T_TripleTask_DroppedTokens, mean (SD) | 0 (0.2) | 0 (0) | 0.1 (0.3) |
| C3T_TripleTask_Attempted, (yes; no), n (%) | 25:1 (96) | 13:0 (100) | 12:1(92) |
| C3T_TripleTask_PassFail, (pass; fail), n (%) | 25:0 (100) | 13:0 (100) | 12:0 (100) |
| **Questionnaires** |  |  |  |
| **IPAQ** |  |  |  |
| IPAQ__Vigorousminsperweek, mean (SD) | 955.1 (2722) | 673.2 (1279.1) | 1236.9 (3692.0) |
| IPAQ_Moderateminsperweek, mean (SD) | 644.8 (1491.2) | 440 (1027.6) | 833.9 (1843.6) |
| IPAQ_Walkingminsperweek, mean (SD) | 2370.8 (3563.7) | 2186.3 (2318.1) | 2541.2 (4517.6) |
| IPAQ_SittingMinsperweek, mean (SD) | 2517.5 (1812) | 2205 (1467.3) | 2830 (2121.1) |
| IPAQ_Total, mean (SD) | 3980.5 (5798.2) | 3191.4 (2444.6) | 4708.9 (7782.2)( |
| **Life Space Assessment** |  |  |  |
| LifeSpace_Level1, mean (SD) | 7.7 (1.1) | 8 (0) | 7.4 (1.5) |
| LifeSpace_Level2, mean (SD) | 14.5 (3.8) | 15.1 (2.4) | 13.8 (4.8) |
| LifeSpace_Level3, mean (SD) | 21.5 (5.1) | 22.2 (3.8) | 20.8 (6.3) |
| LifeSpace_Level4, mean (SD) | 25.4 (8.8) | 28.9 (6.1) | 21.8 (9.9) |
| LifeSpace_Level5, mean (SD) | 21.0 (12.2) | 23.1 (11.1) | 19 (13.3) |
| LifeSpace_Total, mean (SD) | 90.0 (24.7) | 97.2 (16.1) | 82.8 (30.1) |
| **HDProTriad** |  |  |  |
| HDProTriad_CogRaw, mean (SD) | 55 (11.1) | 59.25 (8.8) | 51.1 (11.9) |
| HDProTriad_Cog_Final, mean (SD) | 2.1 (0.8) | 1.7 (0.5) | 2.4 (0.8) |
| HDProTriad_EmotionRaw, mean (SD) | 28.2 (8.5) | 24.3 (6.9) | 31.9 (8.5) |
| HDProTriad_Emotion_Final, mean (SD) | 2.0 (0.6) | 1.7 (0.5) | 2.3 (0.6) |
| HDProTriad_Motor_RawA, mean (SD) | 21.2 (8.6) | 17.1 (3.1) | 25.0 (10.3) |
| HDProTriad_Motor_RawB, mean (SD) | 5.2 (2.4) | 4.2 (0.6) | 6.2 (3.1) |
| HDProTriad_Motor_Final, mean (SD) | 1.4 (0.6) | 1.2 (0.2) | 1.7 (0.7) |
| HDProTriad_Total, mean (SD) | 5.5 (1.4) | 4.6 (0.9) | 6.3 (1.4) |
| **Hospital Anxiety and Depression Score** |  |  |  |
| HADS_Anxiety, mean (SD) | 5.6 (3.0) | 5.3 (2.4) | 5.9 (3.5) |
| HADS_Depression, mean (SD) | 2.5 (2.3) | 2.4 (2.0) | 2.6 (2.6) |
| **Socio-demographic questionnaire** |  |  |  |
| Age (y), mean (SD) | 45.8 (11.1) | 45.6 (11.6) | 45.9 (11.2) |
| Gender (male; female), n (%) | 13:13 (50) | 7:6 (54) | 6:7 (46) |
| Ethnicity  1 = White (n =25)  2 = Mixed / multiple ethnic groups (n=0)  3 = Asian/ British Asian (n=1)  4 = Black/ African/ Caribbean/ Black British (n=0)  5 = Other ethnic group (n=0)  6 = Prefer not to say (n=0) | 1=25 (96)  1 = 3 (4) | 1=13 (100) | 1 = 12(92)  3 = 1 (8) |
| Height (cm), mean (SD) | 169.5 (12.2) | 170.3 (15.0) | 168.7 (9.1) |
| Weight (kg), mean (SD) | 80.7 (19.0) | 79.0 (23.0) | 82.5 (14.6) |
| Drinks Alcohol, (yes; no), n (%) | 19:7 (73) | 10:3 (77) | 9:4 (69) |
| Alcohol Units, (units per week), mean (SD) | 5.7 (6.7) | 6.9 (8.1) | 4.6 (5.0) |
| Smoker, (yes; no), n (%) | 21:5 (81) | 11:2 (85) | 10:3 (77) |
| Cigarettes per day, mean (SD) | 2.0 (5.1) | 0.8 (2.8) | 3.3 (6.6) |
| Smoking years, mean (SD) | 3.5 (8.1) | 2.4 (6.9) | 4.5 (9.3) |
| Caffeine, (yes; no), n (%) | 23:3 (88) | 13:0 (100) | 10:3 (77) |
| Caffeine per day, mean (SD) | 3.7 (2.4) | 3.8 (2.8) | 3.5 (2.0) |
| Handedness, (R; L), n (%) | 22:4 (85) | 11:2 (85 | 11:2 (85) |
| Level of Education (category, n, (%)  1 = No education (n=1)  2 = Entry Level Qualifications (n=1)  3 = GCSEs/O-Levels (n=7)  4 = AS/A-Levels (n=3)  5 = Higher National Diploma or Certificate (n=4)  6 = Degree (n=4)  7 = Postgraduate certificate/diploma or Masters (n=6)  8 = Doctorate (n=0) | 1 = 1 (4)  2 = 1 (4)  3 = 7 (27)  4 = 3 (12)  5 =4 (15)  6 = 4 (15)  7 =6 (23)  8 = 0 (0) | 1= 1 (8)  2= 0 (0)  3=5 (38)  4= 0 (0)  5 = 0 (0)  6 =3 (23)  7 =4 (31)  8 = 0 (0) | 1 = 0 (0)  2 = 1 (8)  3 = 2 (15)  4 = 3 (23)  5 = 4 (31)  6 = 1 (8)  7 = 2 (15)  8 = 0 (0) |
| Years spent in education | 14.9 (4.9) | 14.4 (5.4) | 15.4(4.6) |
| Occupation (Free Text Category) |  |  |  |
| **Cognitive Tests** |  |  |  |
| **Categorical Verbal Fluency** |  |  |  |
| CVF_1, mean (SD) | 7.6 (2.4) | 7.7 (2.4) | 7.5 (2.5) |
| CVF_2, mean (SD) | 4.5 (2.6) | 4.9 (2.7) | 4.1 (2.4) |
| CVF_3, mean (SD) | 3.4 (1.9) | 3.7 (2.3) | 3.2 (1.5) |
| CVF_4, mean (SD) | 2.6 (1.9) | 3.1 (2.1) | 2.2 (1.6) |
| CVF_Total, mean (SD) | 18.1 (6.2) | 19.4 (7.0) | 16.8 (5.3) |
| CVF_Intrusions, mean (SD) | 0 (0) | 0 (0) | 0 (0) |
| CVF_Persev, mean (SD) | 0.8 (1.4) | 0.5 (1.0) | 1.2 (1.8) |
| **Stroop Test** |  |  |  |
| Stroop_WR_Score, mean (SD) | 85.2 (24.3) | 92.2 (23.2) | 78.1 (24.1) |
| Stroop_WR_Error, mean (SD) | 0 (0.1) | 0.1 (0.3) | 0 (0) |
| Stroop_WR_SCError, mean (SD) | 0.1 (0.4) | 0.2 (0.6) | 0 (0) |
| Stroop_CN_Score, mean (SD) | 58.1 (19.2) | 62.8 (15.8) | 53.4 (21.7) |
| Stroop_CN_Errors, mean (SD) | 0 (0) | 0 (0) | 0 (0) |
| Stroop_CN_SCErrors, mean (SD) | 0.4 (0.6) | 0.3 (0.5) | 0.5 (0.8) |
| Stroop_I_Total, mean (SD) | 38.2 (14.8) | 42.2 (15.0) | 34.2 (14.1) |
| Stroop_I_Errors, mean (SD) | 0.5 (1.4) | 0.5 (1.1) | 0.5 (1.7) |
| Stroop_I_SCErrors, mean (SD) | 1.0 (1.2) | 0.8 (0.9) | 1.2 (1.4) |
| **Trail Making Test** |  |  |  |
| TrailMakingA_Time, mean (SD) | 43.2 (54.4) | 33.6 (16.8) | 52.9 (75.4) |
| TrailMakingA_Correct, mean (SD) | 25 (0) | 25 (0) | 25 (0) |
| TrailMakingA_Errors, mean (SD) | 0 (0) | 0 (0) | 0 (0) |
| TrailMakingA_SC_Error, mean (SD) | 0.2 (0.5) | 0.4 (0.7) | 0 (0.2) |
| TrailMakingB_Time, mean (SD) | 76.8 (49.1) | 78.0 (50.8) | 75.5 (50.0) |
| TrailMakingB_Correct, mean (SD) | 24.8 (0.5) | 24.7 (0.6) | 24.8 (0.4) |
| TrailMakingB_Errors, mean (SD) | 0.2 (0.5) | 0.3 (0.6) | 0.2 (0.4) |
| TrailMakingB_SC_Errors, mean (SD) | 0.5 (0.7) | 0.5 (0.7) | 0.6 (0.8) |
| **Letter Verbal Fluency** |  |  |  |
| LVF_F_1, mean (SD) | 4.8 (2.0) | 4.9 (2.1) | 4.8 (2.0) |
| LVF_F_2, mean (SD) | 2.2 (1.6) | 2.4 (1.9) | 2.1 (1.3) |
| LVF_F_3, mean (SD) | 1.7 (1.5) | 1.3 (0.9) | 2.0 (2.0) |
| LVF_F_4, mean (SD) | 1.6 (1.6) | 0.9 (1.1) | 2.3 (1.7) |
| LVF_F_Total, mean (SD) | 10.3 (4.9) | 9.5 (4.7) | 11.2 (5.2) |
| LVF_F_Intrusions, mean (SD) | 0.15 (0.5) | 0.2 (0.6) | 0 (0.2) |
| LVF_F_Persev, mean (SD) | 0.6 (1.0) | 0.3 (0.5) | 0.8 (1.3) |
| LVF_A_1, mean (SD) | 3.8 (1.2) | 3.8 (0.9) | 3.8 (1.5) |
| LVF_A_2, mean (SD) | 1.5 (1.4 ) | 1.8 (1.6) | 1.3 (1.2) |
| LVF_A_3, mean (SD) | 1.0 (1.3) | 1.5 (1.1) | 1.0 (1.3) |
| LVF_A_4, mean (SD) | 7.5 (3.7) | 8.1 (3.8) | 7.0 (3.7) |
| LVF_A_Total, mean (SD) | 7.5 (3.7) | 8.1 (3.8) | 7.0 (3.7) |
| LVF_A_Intrusions, mean (SD) | 0.5 (0.9) | 0.3 (0.5) | 0.7 (1.2) |
| LVF_A_Persev, mean (SD) | 0.4 (0.8) | 0.4 (0.8) | 0.5 (0.9) |
| LVF_S_1, mean (SD) | 4.8 (1.5) | 4.9 (1.4) | 4.7 (1.7) |
| LVF_S_2, mean (SD) | 2.7 (1.6) | 2.6 (1.6) | 2.8 (1.6) |
| LVF_S_3, mean (SD) | 2.3 (1.6) | 2.5 (1.8) | 2.0 (1.5) |
| LVF_S_4, mean (SD) | 1.7 (1.3) | 1.8 (1.5) | 1.6 (1.1) |
| LVF_S_Total, mean (SD) | 11.3 (4.8) | 11.9 (4.3) | 10.7 (5.3) |
| LVF_S_Intrusions, mean (SD) | 0.4 (0.8) | 0.5 (0.7) | 0.3 (0.9) |
| LVF_S_Persev, mean (SD) | 0.5 (0.9) | 0.5 (0.7) | 0.5 (1.1) |
| **Symbol Digit Modality Test** |  |  |  |
| SDMT_TotalCorrect, mean (SD) | 41.4 (16.8) | 44.8 (15.9) | 37.9 (17.5) |
| SDMTT_SC_Error, mean (SD) | 0.2 (0.6) | 0.0 (0.2) | 0.2 (0.8) |
| SDMT_Incorrect, mean (SD) | 0.81 (2.2) | 0.3 (0.6) | 1.3 (3.1) |

**Table 2– Descriptive data for home visit assessments at home visit one.**

| Home Visits Assessments | Overall  (n=26) | Control Group  (n=13) | Intervention Group  (n=13) |
| --- | --- | --- | --- |
| **Cognitive Tests** |  |  |  |
| **Categorical Verbal Fluency** |  |  |  |
| CVF_1, mean (SD) | 7.7 (2.6) | 8.2 (2.7) | 7.2 (2.4) |
| CVF_2, mean (SD) | 4.5 (2.1) | 4.0 (2.1) | 5.1 (2.1) |
| CVF_3, mean (SD) | 3.8 (1.8) | 4.0 (2.1) | 3.6 (1.4) |
| CVF_4, mean (SD) | 2.6 (1.9) | 2.2 (1.7) | 3.0 (2.1) |
| CVF_Total, mean (SD) | 18.7 (5.6) | 18.5 (6.0) | 18.9 (5.3) |
| CVF_Intrusions, mean (SD) | 0 (0) | 0 (0) | 0 (0) |
| CVF_Persev, mean (SD) | 0.5 (0.8) | 0.7 (0.9) | 0.3 (0.5) |
| **Stroop Test** |  |  |  |
| Stroop_WR_Score, mean (SD) | 85.4 (27.3) | 90.8 (23.9) | 80.0 (30.3) |
| Stroop_WR_Error, mean (SD) | 0.2 (0.5) | 0.2 (0.4) | 0.2 (0.6) |
| Stroop_WR_SCError, mean (SD) | 0 (0.3) | 0.2 (0.4) | 0 (0) |
| Stroop_CN_Score, mean (SD) | 595.9 (19.0) | 64.5 (15.0) | 55.3 (21.9) |
| Stroop_CN_Errors, mean (SD) | 0.5 (0.6) | 0.6 (0.7) | 0.3 (0.5) |
| Stroop_CN_SCErrors, mean (SD) | 0 (0.2) | 0 (0) | 0 (0.3) |
| Stroop_I_Total, mean (SD) | 38.3 (13.7) | 41.6 (13.3) | 35.0 (13.8) |
| Stroop_I_Errors, mean (SD) | 1.4 (1.5) | 1 .0 (0.9) | 1.8 (1.9) |
| Stroop_I_SCErrors, mean (SD) | 0.1 (0.4) | 0.2 (0.6) | 0 (0.2) |
| **Trail Making Test** |  |  |  |
| TrailMakingA_Time, mean (SD) | 38.3 (46.2) | 28.9 (16.6) | 47.7 (63.1) |
| TrailMakingA_Correct, mean (SD) | 25 (0) | 25 (0) | 25 (0) |
| TrailMakingA_Errors, mean (SD) | 0 (0) | 0 (0) | 0 (0) |
| TrailMakingA_SC_Error, mean (SD) | 0.1 (0.3) | 0 (0.2) | 0.2 (0.4) |
| TrailMakingB_Time, mean (SD) | 63.5 (36.7) | 65.3 (41.1) | 61.6 (32.9) |
| TrailMakingB_Correct, mean (SD) | 25.0 (0.2) | 25 (0) | 24.9 (0.3) |
| TrailMakingB_Errors, mean (SD) | 0.1 (0.3) | 0 (0) | 0.2 (0.4) |
| TrailMakingB_SC_Errors, mean (SD) | 0.2 (0.4) | 0.3 (0.5) | 0.2 (0.4) |
| **Letter Verbal Fluency** |  |  |  |
| LVF_F_1, mean (SD) | 5.1 (2.2) | 5.5 (2.0) | 4.7 (2.4) |
| LVF_F_2, mean (SD) | 2.5 (1.5) | 1.6 (1.0) | 3.2 (1.6) |
| LVF_F_3, mean (SD) | 2.4 (1.3) | 2.4 (1.1) | 2.5 (1.5) |
| LVF_F_4, mean (SD) | 1.5 (1.3) | 1.4 (1.1) | 1.6 (1.4) |
| LVF_F_Total, mean (SD) | 11.5 (4.8) | 10.9 (4.0) | 11.9 (5.5) |
| LVF_F_Intrusions, mean (SD) | 0.5 (0.7) | 0.5 (0.7) | 0.4 (0.7) |
| LVF_F_Persev, mean (SD) | 0.8 (1.1) | 0.3 (0.5) | 1.2 (1.3) |
| LVF_A_1, mean (SD) | 3.9 (1.4) | 4.2 (1.3) | 3.6 (1.6) |
| LVF_A_2, mean (SD) | 2.0 (1.4) | 1.5 (1.2) | 2.3 (1.5) |
| LVF_A_3, mean (SD) | 1.8 (1.8) | 1.5 (1.7) | 2.0 (1.9) |
| LVF_A_4, mean (SD) | 1.4 (1.2) | 1.4 (1.1) | 1.4 (1.3) |
| LVF_A_Total, mean (SD) | 9.0 (4.6) | 8.5 (4.0) | 9.3 (5.2) |
| LVF_A_Intrusions, mean (SD) | 0.6 (0.8) | 0.5 (0.7) | 0.7 (0.9) |
| LVF_A_Persev, mean (SD) | 0.3 (0.8) | 0.1 (0.3) | 0.5 (1.0) |
| LVF_S_1, mean (SD) | 5.2 (1.6) | 5.3 (1.9) | 5.2 (1.5) |
| LVF_S_2, mean (SD) | 2.9 (1.7) | 2.7 (1.7) | 3.0 (1.7) |
| LVF_S_3, mean (SD) | 2.3 (1.5) | 2.1 (1.6) | 2.4 (1.4) |
| LVF_S_4, mean (SD) | 2.1 (1.3) | 2.5 (1.2) | 1.8 (1.4) |
| LVF_S_Total, mean (SD) | 12.4 (4.6) | 12.5 (4.8) | 12.3 (4.6) |
| LVF_S_Intrusions, mean (SD) | 0.2 (0.4) | 0.1 (0.3) | 0.2 (0.4) |
| LVF_S_Persev, mean (SD) | 0.5 (0.7) | 0.4 (0.5) | 0.5 (0.8) |
| **Digit Span** |  |  |  |
| DigitSpan_TotalSequences | 15.0 (3.0) | 15.2 (4.0) | 14.9 (2.0) |
| DigitSpan_max_span, mean (SD) | 6.2 (1.1) | 6.2 (1.4) | 6.2 (0.7) |
| DigitSpan_3correct, mean (SD) | 5.2 (1.1) | 5.4 (1.5) | 5.1 (0.6) |
| DualTask_digitspan_correct_single, mean (SD) | 10.8 (5.0) | 11.2 (5.1) | 10.4 (5.0) |
| DualTask_digitspan_incorrect_single, mean (SD) | 3.3 (2.5) | 3.2 (2.6) | 3.4 (2.4) |
| DualTask_digitspan_correct_double, mean (SD) | 11.3 (5.6) | 12.2 (5.7) | 10.4 (5.6) |
| DualTask_digitspan_incorrect_double, mean (SD) | 2.4 (2.6) | 2.0 (2.7) | 2.8 (2.6) |
| DualTask_boxes_single, mean (SD) | 125.7 (46.8) | 131.0 (43.5) | 120.3 (51.0) |
| DualTask_boxes_double, mean (SD) | 117.0 (45.5) | 127.5 (34.6) | 106.4 (53.5) |
| **Tower of Hanoi** |  |  |  |
| ToH_TotalItemsCorrect, mean (SD) | 7.5 (1.7) | 7.6 (1.4) | 7.3 (2.1) |
| ToH_TotalRawScore, mean (SD) | 16.7 (4.6) | 17.1 (3.6) | 16.4 (5.6) |
| ToH_ScaledScore, mean (SD) | 10.3 (2.9) | 10.5 (2.5) | 10.0 (3.4) |
| ToH_TimePerMoveRatio, mean (SD) | 4.0 (2.0) | 3.8 (1.6) | 4.3 (2.4) |
| ToH_TimePerMoveRatio_Scaled, mean (SD) | 8.9 (3.7) | 9.4 (2.7) | 8.4 (4.5) |
| ToH_MoveAccuracyRatio, mean (SD) | 1.9 (3.1) | 2.5 (4.4) | 1.4 (0.3) |
| ToH_MoveAccuracyRatio_Scaled, mean (SD) | 10.9 (1.8) | 10.9 (2.0) | 10.8 (1.7) |
| ToH_TotalRuleViolations, mean (SD) | 1.0 (2.5) | 1.4 (3.3) | 0.6 (1.3) |
| ToH_TotalRuleViolations_CPR, mean (SD) | 80.5 (35.2) | 77.4 (37.8) | 83.7 (33.5) |
| ToH_RuleViolationPerItemRatio_scaled, mean (SD) | 9.9 (2.5) | 9.4 (3.3) | 10.5 (1.2) |
| **Card Sorting Test** |  |  |  |
| Sort_WordsIncorrectlyRead, mean (SD) | 0 (0) | 0 (0) | 0 (0) |
| Sort_WordsNotUnderstood, mean (SD) | 0 (0) | 0 (0) | 0 (0) |
| Sort_TotalDescriptionScore, mean (SD) | 15.4 (6.6) | 16.1 (6.0) | 14.8 (7.3) |
| Sort_NumberCorrectConfirmedSorts, mean (SD) | 3.9 (1.6) | 4.0 (1.5) | 3.8 (1.7) |
| Sort_CumulativeSortingTime, mean (SD) | 70.3 (47.1) | 68.7 (55.8) | 71.9 (38.8) |
| **Symbol Digit Modality Test** |  |  |  |
| SDMT_TotalCorrect, mean (SD) | 42.1 (19.1) | 47.9 (19.2) | 36.2 (17.8) |
| SDMTT_SC_Error, mean (SD) | 1.0 (2.0) | 0.4 (0.9) | 1.5 (2.7) |
| SDMT_Incorrect, mean (SD) | 0.2 (0.4) | 0.2 (0.4) | 0.2 (0.4) |

**Table 3 – Descriptive data of home visit assessments for home visit two.**

| Home Visits Assessments | Overall  (n=24) | Control Group  (n=13) | Intervention Group  (n=11) |
| --- | --- | --- | --- |
| **Cognitive Tests** |  |  |  |
| **Categorical Verbal Fluency** |  |  |  |
| CVF_1, mean (SD) | 7.8 (3.0) | 9.0 (2.9) | 6.5 (2.5) |
| CVF_2, mean (SD) | 4.4 (2.6) | 4.0 (2.5) | 4.8 (2.7) |
| CVF_3, mean (SD) | 3.1 (2.0) | 3.2 (2.0) | 3.1 (2.2) |
| CVF_4, mean (SD) | 2.5 (2.1) | 3.2 (2.5) | 1.8 (1.1) |
| CVF_Total, mean (SD) | 17.9 (6.7) | 19.3 (7.4) | 16.2 (5.7) |
| CVF_Intrusions, mean (SD) | 0 (0) | 0 (0) | 0 (0) |
| CVF_Persev, mean (SD) | 1.1 (1.8) | 0.8 (1.4) | 1.5 (2.2) |
| **Stroop Test** |  |  |  |
| Stroop_WR_Score, mean (SD) | 85.3 (27.7) | 91.8 (21.5) | 77.6 (32.9) |
| Stroop_WR_Error, mean (SD) | 0.2 (0.4) | 0.2 (0.4) | 0.3 (0.5) |
| Stroop_WR_SCError, mean (SD) | 0 (0) | 0 (0) | 0 (0) |
| Stroop_CN_Score, mean (SD) | 60.2 (20.3) | 67.4 (17.7) | 51.7 (20.7) |
| Stroop_CN_Errors, mean (SD) | 0.8 (0.9) | 0.8 (1.0) | 0.7 (0.9) |
| Stroop_CN_SCErrors, mean (SD) | 0 (0.4) | 0 (0) | 0.2 (0.6) |
| Stroop_I_Total, mean (SD) | 38.4 (15.7) | 43.1 (15.8) | 32.9 (14.3) |
| Stroop_I_Errors, mean (SD) | 1.3 (1.4) | 1.3 (1.1) | 1.3 (1.7) |
| Stroop_I_SCErrors, mean (SD) | 0.5 (1.9) | 0.7 (2.5) | 0.3 (0.6) |
| **Trail Making Test** |  |  |  |
| TrailMakingA_Time, mean (SD) | 36.7 (33.2) | 29.5 (16.8) | 45.2 (45.2) |
| TrailMakingA_Correct, mean (SD) | 25 (0) | 25 (0) | 25 (0) |
| TrailMakingA_Errors, mean (SD) | 0 (0.2) | 0 (0) | 0.1 (0.3) |
| TrailMakingA_SC_Error, mean (SD) | 0.1 (0.3) | 0.2 (0.4) | 0 (0) |
| TrailMakingB_Time, mean (SD) | 71.5(53.7) | 55.4 (44.4) | 92.5 (59.6) |
| TrailMakingB_Correct, mean (SD) | 24.4 (2.3) | 24.9 (0.3) | 23.7 (3.4) |
| TrailMakingB_Errors, mean (SD) | 0.2 (0.4) | 0.1 (0.3) | 0.3 (0.5) |
| TrailMakingB_SC_Errors, mean (SD) | 0.6 (0.8) | 0.2 (0.4) | 1.2 (0.9) |
| **Letter Verbal Fluency** |  |  |  |
| LVF_F_1, mean (SD) | 5.3 (1.9) | 5.8 (1.9) | 4.7 (1.8) |
| LVF_F_2, mean (SD) | 2.6 (1.7) | 2.4 (1.6) | 2.8 (1.8) |
| LVF_F_3, mean (SD) | 2.3 (1.3) | 2.5 (1.5) | 2.2 (1.0) |
| LVF_F_4, mean (SD) | 1.9 (1.5) | 1.6 (1.4) | 2.2 (1.7) |
| LVF_F_Total, mean (SD) | 12.1 (4.1) | 12.3 (4.2) | 11.9 (4.3) |
| LVF_F_Intrusions, mean (SD) | 0.6 (0.9) | 0.6 (1.0) | 0.5 (0.8) |
| LVF_F_Persev, mean (SD) | 0.7 (0.9) | 0.5 (0.8) | 0.9 (1.0) |
| LVF_A_1, mean (SD) | 4.2 (1.6) | 4.8 (1.2) | 3.6 (1.8) |
| LVF_A_2, mean (SD) | 2.0 (1.4) | 1.8 (1.3) | 2.3 (1.6) |
| LVF_A_3, mean (SD) | 1.3 (1.3) | 1.0 (1.1) | 1.7 (1.5) |
| LVF_A_4, mean (SD) | 1.3 (1.3) | 1.6 (1.4) | 1.1 (1.0) |
| LVF_A_Total, mean (SD) | 9.7 (4.6) | 10.5 (4.8) | 8.7 (4.3) |
| LVF_A_Intrusions, mean (SD) | 0.3 (0.7) | 0.3 (0.5) | 0.5 (0.9) |
| LVF_A_Persev, mean (SD) | 0.7 (0.9) | 0.5 (0.8) | 0.8 (1.0) |
| LVF_S_1, mean (SD) | 5.1 (1.6) | 5.6 (1.3) | 4.5 (1.7) |
| LVF_S_2, mean (SD) | 3.5 (2.2) | 3.3 (1.4) | 3.7 (2.8) |
| LVF_S_3, mean (SD) | 2.7 (1.5) | 2.8 (1.3) | 2.5 (1.7) |
| LVF_S_4, mean (SD) | 2.6 (1.5) | 2.2 (1.5) | 3.0 (1.5) |
| LVF_S_Total, mean (SD) | 13.8 (4.7) | 13.8 (4.1) | 13.7 (5.5) |
| LVF_S_Intrusions, mean (SD) | 0.7 (1.6) | 0.7 (2.0) | 0.7 (1.1) |
| LVF_S_Persev, mean (SD) | 0.4 (0.9) | 0.3 (0.9) | 0.5 (0.9) |
| **Digit Span** |  |  |  |
| DigitSpan_MaxSequences | 14.7 (3.2) | 15.2 (4.0) | 14.0 (2.1) |
| DigitSpan_max_span, mean (SD) | 6.0 (1.0) | 6.2 (1.3) | 5.7 (0.6) |
| DigitSpan_3correct, mean (SD) | 5.2 (1.0) | 5.3 (1.3) | 5.0 (0.8) |
| DualTask_digitspan_correct_single, mean (SD) | 11.9 (5.7) | 11.0 (5.8) | 13.1 (5.7) |
| DualTask_digitspan_incorrect_single, mean (SD) | 3.1 (2.8) | 3.6 (3.0) | 2.5 (2.4) |
| DualTask_digitspan_correct_double, mean (SD) | 11.8 (5.5) | 11.8 (5.9) | 11.8 (5.3) |
| DualTask_digitspan_incorrect_double, mean (SD) | 3.1 (2.6) | 3.0 (3.0) | 3.3 (2.1) |
| DualTask_boxes_single, mean (SD) | 128.6 (45.7) | 137.1 (38.1) | 118.6 (53.6) |
| DualTask_boxes_double, mean (SD) | 115.6 (52.0) | 133.8 (41.2) | 94.1 (56.8) |
| **Tower of Hanoi** |  |  |  |
| ToH_TotalItemsCorrect, mean (SD) | 8.0 (1.4) | 7.9 (1.5) | 8.0 (1.3) |
| ToH_TotalRawScore, mean (SD) | 19.8 (6.2) | 17.8 (5.6) | 22.0 (6.3) |
| ToH_ScaledScore, mean (SD) | 12.6 (3.7) | 11.5 (2.9) | 13.9 (4.2) |
| ToH_TimePerMoveRatio, mean (SD) | 4.7 (4.4) | 4.7 (3.8) | 4.7 (5.3) |
| ToH_TimePerMoveRatio_Scaled, mean (SD) | 9.3 (3.5) | 9.0 (3.3) | 9.6 (3.8) |
| ToH_MoveAccuracyRatio, mean (SD) | 1.4 (0.4) | 1.5 (0.5) | 1.2 (0.3) |
| ToH_MoveAccuracyRatio_Scaled, mean (SD) | 11.3 (1.8) | 10.7 (1.5) | 12.1 (1.9) |
| ToH_TotalRuleViolations, mean (SD) | 0.2 (0.8) | 0 (0) | 0.5 (1.2) |
| ToH_TotalRuleViolations_CPR, mean (SD) | 90.0 (29.0) | 92.3 (27.7) | 86.5 (31.0) |
| ToH_RuleViolationPerItemRatio_scaled, mean (SD) | 10.5 (2.1) | 10.9 (0.3) | 10.0 (3.2) |
| **Card Sorting Test** |  |  |  |
| Sort_WordsIncorrectlyRead, mean (SD) | 0 (0) | 0 (0) | 0 (0) |
| Sort_WordsNotUnderstood, mean (SD) | 0 (0) | 0 (0) | 0 (0) |
| Sort_TotalDescriptionScore, mean (SD) | 16.1 (5.1) | 17.1 (3.3) | 14.9 (6.7) |
| Sort_NumberCorrectConfirmedSorts, mean (SD) | 4.3 (1.3) | 4.7 (0.6) | 3.7 (1.7) |
| Sort_CumulativeSortingTime, mean (SD) | 92.6 (60.7) | 84.0 (50.6) | 102.6 (72.2) |
| **Symbol Digit Modality Test** |  |  |  |
| SDMT_TotalCorrect, mean (SD) | 40.8 (16.9) | 43.8 (17.0) | 37.2 (16.7) |
| SDMT_Incorrect, mean (SD) | 0.7 (1.2) | 0.8 (1.4) | 0.5 (1.0) |

**Table 4 - Descriptive data for all outcome measurements:**

| Outcome Measures | Overall  (n=23) | Control Group  (n=13) | Intervention Group  (n=10) |
| --- | --- | --- | --- |
| **Motor Tasks** |  |  |  |
| **Timed Up and Go** |  |  |  |
| TUG_Baseline_Time To Rise, mean (SD) | 0.87 (0.3) | 0.9 (0.4) | 0.8 (0.2) |
| TUG_Baseline_Time To Walk, mean (SD) | 8.2 (1.8) | 8.0 (1.9) | 8.5 (1.9) |
| TUG_Baseline_Time To Complete, mean (SD) | 9.1 (1.8) | 8.9 (1.8) | 9.4 (2.0) |
| Alphabet_Baseline, mean (SD) | 7.0 (3.3) | 6.8 (3.3) | 7.2 (3.5) |
| TUG_Motor_Time To Rise, mean (SD) | 1.0 (0.7) | 0.9 (0.4) | 1.2 (1.1) |
| TUG_Motor_Time To Walk, mean (SD) | 8.5 (2.2) | 8.2 (2.3) | 8.8 (2.0) |
| TUG_Motor_Time To Complete, mean (SD) | 9.6 (2.3) | 9.5 (2.4) | 9.8 (2.3) |
| TUG_Cog_Time To Rise, mean (SD) | 0.7 (0.3) | 0.8 (0.3) | 0.6 (0.1) |
| TUG_Cog_Time To Walk, mean (SD) | 7.8 (2.0) | 7.8 (2.4) | 7.8 (1.0) |
| TUG_Cog_Time To Complete, mean (SD) | 8.7 (2.0) | 8.8 (2.4) | 8.5 (1.1) |
| TUG_Triple_Time To Rise, mean (SD) | 0.8 (0.2) | 0.8 (0.2) | 0.7 (0.1) |
| TUG_Triple_Time To Walk, mean (SD) | 9.2 (3.4) | 8.9 (3.1) | 9.6 (4.0) |
| TUG_Triple_Time To Complete, mean (SD) | 10.5 (4.6) | 9.9 (3.4) | 11.3 (6.1) |
| **Quantitative Timed Up and Go** |  |  |  |
| QTUG_Baseline_Time To Complete, mean (SD) | 9.5 (2.1) | 9.4 (2.4) | 9.5 (1.7) |
| QTUG_Baseline_Time To Stand, mean (SD) | 1.1 (0.5) | 1.0 (0.3) | 1.1 (0.6) |
| QTUG_Baseline_stride, mean (SD) | 1.2 (0.1) | 1.1 (0.1) | 1.2 (0.2) |
| QTUG_Baseline_velocity, mean (SD) | 130.8 (24.7) | 131.6 (28.4) | 130.0 (20.5) |
| QTUG_Baseline_Time To Turn, mean (SD) | 2.4 (1.1) | 2.5 (1.3) | 2.3 (0.7) |
| QTUG_Baseline_STT, , mean (SD) | 1.8 (0.8) | 1.8 (0.8) | 1.9 (0.8) |
| QTUG_Motor_Time to Stand, mean (SD) | 9.7 (2.5) | 9.5 (2.6) | 9.9 (2.5) |
| QTUG_Motor_Time To Complete, mean (SD) | 1.1 (0.5) | 0.9 (0.4) | 1.3 (0.6) |
| QTUG_Motor_stride, mean (SD) | 1.2 (0.2) | 1.1 (0.2) | 1.3 (0.3) |
| QTUG_Motor_velocity, mean (SD) | 129.0 (24.9) | 131.7 (25.4) | 125.5 (25.4) |
| QTUG_Motor_Time To Turn, mean (SD) | 2.0 (0.7) | 1.8 (0.6) | 2.3 (0.8) |
| QTUG_Motor_STT, mean (SD) | 1.7 (0.8) | 1.7 (0.6) | 1.8 (1.0) |
| QTUG_Cog_Time To Complete, mean (SD) | 9.0 (2.2) | 9.2 (2.7) | 8.6 (1.0) |
| QTUG_Cog_Time To Stand, mean (SD) | 0.8 (0.3) | 0.85 (0.2) | 0.6 (0.3) |
| QTUG_Cog_stride, mean (SD) | 1.2 (0.2) | 1.1 (0.2) | 1.2 (0.2) |
| QTUG_Cog_velocity, mean (SD) | 130.2 (28.6) | 132.5 (32.7) | 126.6 (22.5) |
| QTUG_Cog_Time to turn, mean (SD) | 2.8 (1.1) | 2.6 (1.1) | 3.1 (1.0) |
| QTUG_Cog_STT, mean (SD) | 2.1 (1.0) | 2.2 (1.1) | 1.9 (0.9) |
| QTUG_Dual_Time to complete, mean (SD) | 10.6 (5.0) | 9.9 (3.8) | 11.6 (6.4) |
| QTUG_Dual_Time to sit, mean (SD) | 0.9 (0.4) | 0.9 (0.5) | 0.9 (0.3) |
| QTUG_Dual_stride, mean (SD) | 1.2 (0.2) | 1.2 (0.2) | 1.3(0.2) |
| QTUG_Dual_velocity, mean (SD) | 125.1 (28.2) | 128.9 (32.1) | 120.0 (22.7) |
| QTUG_Dual_Time to turn, mean (SD) | 3.3 (2.2) | 2.8 (1.7) | 4.0 (2.6) |
| QTUG_Dual_STT, mean (SD) | 1.8 (0.9) | 1.6 (0.7) | 2.1 (1.1) |
| **Clinch Token Transfer Test (C3T)** |  |  |  |
| C3T_Gender (male; female), n (%) | 12:11 (52%) | 7:6 (54%) | 5:5  (50%) |
| C3T_Handedness (R; L), n (%) | 21:2 (91%) | 13:0 (100%) | 8:2 (80%) |
| C3T_Baseline_TimeTaken, mean (SD) | 34.2 (71.5) | 19.3 (10.1) | 53.5 (107.8) |
| C3T_Baseline_RuleError, mean (SD) | 0.0 (0) | 0.0 (0) | 0.0 (0) |
| C3T_Baseline_DroppedTokens, mean (SD) | 0.0 (0.2) | 0.1 (0.3) | 0.0 (0) |
| C3T_Baseline_TransferError, mean (SD) | 0.0 (0.2) | 0.1 (0.3) | 0.0 (0) |
| C3T_TokenValue_Baseline_TimeTaken, mean (SD) | 7.1 (4.1) | 6.6 (3.0) | 7.8 (5.5) |
| C3T_TokenValue_Baseline_Attempted, (yes; no), n (%) | 22:1 (96%) | 13:0 (100%) | 9:1  (90%) |
| C3T_TokenValue_Baseline_PassFail, (pass; fail), n (%) | 22:1 (96%) | 13:0 (100%) | 9:1 (90%) |
| C3T_Alphabet_Baseline_TimeTaken, mean (SD) | 7.6 (3.9) | 7.3 (3.4) | 8.2 (4.5) |
| C3T_Alphabet_Baseline_CorrectAnswers, mean (SD) | 26 (0) | 26 (0) | 26 (0) |
| C3T_Alphabet_Baseline_Error, mean (SD) | 0.0 (0) | 0.0 (0) | 0.0 (0) |
| C3T_Alphabet_Baseline_Attempted, (yes; no), n (%) | 22:1 (96%) | 13:0 (100%) | 9:1  (90%) |
| C3T_Alphabet_Baseline_PassFail, (pass; fail), n (%) | 22:1 (96%) | 13:0 (100%) | 9:1 (90%) |
| C3T_DualTask_TimeTaken, mean (SD) | 21.2 (8.8) | 19.7 (23.4) | 23.4 (8.8) |
| C3T_DualTask_RuleError, mean (SD) | 0.1 (0.4) | 0.0 (0) | 0.2 (0.7) |
| C3T_DualTask_DroppedTokens, mean (SD) | 0.0 (0) | 0.0 (0) | 0.1 (0.3) |
| C3T_DualTask_TransferError, mean (SD) | 0.0 (0) | 0.0 (0) | 0.0 (0) |
| C3T_DualTask_Attempted, (yes; no), n (%) | 22:1 (96%) | 13:0 (100%) | 9:1  (90%) |
| C3T_DualTask_PassFail, (pass; fail), n (%) | 22:1 (96%) | 13:0 (100%) | 9:1 (90%) |
| C3T_TripleTask_TimeTaken, mean (SD) | 23.1 (10.5) | 22.0 (11.2) | 24.8 (10.0) |
| C3T_TripleTask_AlphabetCorrectAnswers, mean (SD) | 26 (0) | 26 (0) | 26 (0) |
| C3T_TripleTask_Rule_Error, mean (SD) | 0.0 (0) | 0.1 (0.3) | 0.0 (0) |
| C3T_TripleTask_AlphabetErrors, mean (SD) | 0.0 (0) | 0.0 (0) | 0.0 (0) |
| C3T_TripleTask_TransferError, mean (SD) | 0.1 (0.4) | 0.2 (0.4) | 0.1 (0.3) |
| C3T_TripleTask_DroppedTokens, mean (SD) | 0.0 (0.2) | 0.1 (0.3) | 0.0 (0) |
| C3T_TripleTask_Attempted, (yes; no), n (%) | 22:1 (96%) | 13:0 (100%) | 9:1  (90%) |
| C3T_TripleTask_PassFail, (pass; fail), n (%) | 22:1 (96%) | 13:0 (100%) | 9:1  (90%) |
| **Questionnaires** |  |  |  |
| **IPAQ** |  |  |  |
| IPAQ__Vigorousminsperweek, mean (SD) | 1043.5 (2539.5) | 756.9 (1392.0) | 1416.0 (3592.8) |
| IPAQ_Moderateminsperweek, mean (SD) | 957.4 (1576.3) | 627.7 (835.9) | 1386.0 (2186.6) |
| IPAQ_Walkingminsperweek, mean (SD) | 2030.9 (3170.0) | 1832.0 (1973.1) | 2289.5 (4388.0) |
| IPAQ_SittingMinsperweek, mean (SD) | 2109.5 (1464.2) | 1837.5 (1122.4) | 2436.0 (1801.6) |
| IPAQ_Total, mean (SD) | 4029.6 (5405.7) | 3212.8 (3369.0) | 5091.5 (7353.9) |
| **Life Space Assessment** |  |  |  |
| LifeSpace_Level1, mean (SD) | 7.8 (0.9) | 8 (0) | 7.6 (1.3) |
| LifeSpace_Level2, mean (SD) | 15.6 (1.7) | 16 (0) | 15.1 (2.7) |
| LifeSpace_Level3, mean (SD) | 22.0 (4.7) | 22.6 (3.6) | 21 (6.0) |
| LifeSpace_Level4, mean (SD) | 23.7 (10.3) | 24.6 (9.5) | 22.4 (11.7) |
| LifeSpace_Level5, mean (SD) | 22.7 (13.8) | 24.6 (15.1) | 20 (12.0) |
| LifeSpace_Total, mean (SD) | 92.0 (25.7) | 95.8 (22.6) | 86.6 (30.3) |
| **HDProTriad** |  |  |  |
| HDProTriad_CogRaw, mean (SD) | 58.0 (10.9) | 62.3 (6.2) | 52.3 (13.4) |
| HDProTriad_Cog_Final, mean (SD) | 1.9 (0.8) | 1.6 (0.4) | 2.3 (1.0) |
| HDProTriad_EmotionRaw, mean (SD) | 24.9 (8.8) | 23.4 (8.5) | 26.8 (9.3) |
| HDProTriad_Emotion_Final, mean (SD) | 1.8 (0.6) | 1.7 (0.6) | 1.9 (0.7) |
| HDProTriad_Motor_RawA, mean (SD) | 22.1 (11.3) | 17.1 (2.5) | 28.8 (15.0) |
| HDProTriad_Motor_RawB, mean (SD) | 7.7 (5.1) | 7.6 (6.0) | 7.9 (3.8) |
| HDProTriad_Motor_Final, mean (SD) | 1.5 (0.7) | 1.2 (0.2) | 1.9 (1.0) |
| HDProTriad_Total, mean (SD) | 5.1 (1.7) | 4.4 (1.0) | 6.1 (1.9) |
| **Hospital Anxiety and Depression Score** |  |  |  |
| HADS_Anxiety, mean (SD) | 5.0 (3.6) | 4.5 (4.3) | 5.8 (2.4) |
| HADS_Depression, mean (SD) | 1.8 (2.6) | 1.8 (3.1) | 1.8 (1.9) |
| **Cognitive Tests** |  |  |  |
| **Categorical Verbal Fluency** |  |  |  |
| CVF_1, mean (SD) | 7.7 (2.5) | 7.8 (2.3) | 7.5 (2.8) |
| CVF_2, mean (SD) | 5.6 (2.4) | 5.5 (2.8) | 5.7 (2.0) |
| CVF_3, mean (SD) | 3.4 (2.1) | 3.2 (2.1) | 3.8 (2.1) |
| CVF_4, mean (SD) | 2.8 (1.8) | 2.7 (2.3) | 2.9 (0.9) |
| CVF_Total, mean (SD) | 19.5 (6.8) | 19.2 (7.5) | 19.9 (6.2) |
| CVF_Intrusions, mean (SD) | 0.0 (0.2) | 0.1 (0.3) | 0.0 (0) |
| CVF_Persev, mean (SD) | 0.7 (1.2) | 0.5 (0.9) | 1.0 (1.6) |
| **Stroop Test** |  |  |  |
| Stroop_WR_Score, mean (SD) | 83.7 (24.2) | 89.6 (20.2) | 76.1 (27.9) |
| Stroop_WR_Error, mean (SD) | 0.0 (0.2) | 0.1 (0.3) | 0.0 (0) |
| Stroop_WR_SCError, mean (SD) | 0.0 (0) | 0.0 (0) | 0.0 (0) |
| Stroop_CN_Score, mean (SD) | 60.4 (19.8) | 65.7 (15.5) | 53.5 (23.3) |
| Stroop_CN_Errors, mean (SD) | 0.2 (0.5) | 0.2 (0.4) | 0.3 (0.7) |
| Stroop_CN_SCErrors, mean (SD) | 0.7 (1.2) | 0.4 (0.7) | 1.1 (1.7) |
| Stroop_I_Total, mean (SD) | 37.0 (15.0) | 40.7 (14.4) | 32.3 (15.2) |
| Stroop_I_Errors, mean (SD) | 0.7 (1.0) | 0.7 (0.9) | 0.6 (1.1) |
| Stroop_I_SCErrors, mean (SD) | 1.0 (0.9) | 1.2 (1.0) | 0.7 (0.7) |
| **Trail Making Test** |  |  |  |
| TrailMakingA_Time, mean (SD) | 38.3 (45.7) | 29.3 (23.4) | 50.0 (64.1) |
| TrailMakingA_Correct, mean (SD) | 25.0 (0.2) | 25.0 (0.3) | 25.0 (0) |
| TrailMakingA_Errors, mean (SD) | 0.0 (0.2) | 0.1 (0.3) | 0.0 (0) |
| TrailMakingA_SC_Error, mean (SD) | 0.2 (0.5) | 0.0 (0) | 0.5 (0.7) |
| TrailMakingB_Time, mean (SD) | 71.6 (46.8) | 73.1 (53.7) | 69.3 (37.8) |
| TrailMakingB_Correct, mean (SD) | 24.8 (0.5) | 24.9 (0.3) | 24.7 (0.7) |
| TrailMakingB_Errors, mean (SD) | 0.2 (0.5) | 0.1 (0.3) | 0.3 (0.7) |
| TrailMakingB_SC_Errors, mean (SD) | 0.2 (0.4) | 0.2 (0.4) | 0.1 (0.3) |
| **Letter Verbal Fluency** |  |  |  |
| LVF_F_1, mean (SD) | 5.8 (2.4) | 6.3 (2.8) | 5.1 (1.8) |
| LVF_F_2, mean (SD) | 2.8 (1.7) | 2.7 (1.7) | 3.0 (1.8) |
| LVF_F_3, mean (SD) | 2.0 (1.8) | 1.7 (1.6) | 2.4 (2.0) |
| LVF_F_4, mean (SD) | 1.6 (1.3) | 1.3 (1.1) | 2.0 (1.4) |
| LVF_F_Total, mean (SD) | 12.2 (5.2) | 11.9 (4.5) | 12.5 (6.2) |
| LVF_F_Intrusions, mean (SD) | 0.5 (0.7) | 0.3 (0.8) | 0.6 (0.7) |
| LVF_F_Persev, mean (SD) | 0.7 (1.0) | 0.7 (1.2) | 0.8 (0.8) |
| LVF_A_1, mean (SD) | 4.5 (1.7) | 4.6 (1.9) | 4.4 (1.3) |
| LVF_A_2, mean (SD) | 2.7 (1.6) | 2.3 (1.3) | 3.3 (1.8) |
| LVF_A_3, mean (SD) | 1.7 (1.3) | 1.6 (1.1) | 1.9 (1.5) |
| LVF_A_4, mean (SD) | 1.6 (1.2) | 1.3 (1.3) | 2.0 (1.2) |
| LVF_A_Total, mean (SD) | 10.4 (3.8) | 9.5 (3.6) | 11.5 (4.0) |
| LVF_A_Intrusions, mean (SD) | 0.4 (0.7) | 0.3 (0.5) | 0.5 (1.0) |
| LVF_A_Persev, mean (SD) | 0.6 (0.9) | 0.5 (0.8) | 0.7 (1.1) |
| LVF_S_1, mean (SD) | 5.4 (2.1) | 6.2 (1.5) | 4.5 (2.3) |
| LVF_S_2, mean (SD) | 3.1 (1.4) | 3.1 (1.3) | 3.1 (1.6) |
| LVF_S_3, mean (SD) | 2.7 (1.7) | 2.8 (2.1) | 2.7 (1.3) |
| LVF_S_4, mean (SD) | 2.7 (1.7) | 2.3 (1.4) | 3.2 (2.0) |
| LVF_S_Total, mean (SD) | 13.8 (5.0) | 14.0 (4.8) | 13.5 (5.4) |
| LVF_S_Intrusions, mean (SD) | 0.3 (0.6) | 0.1 (0.3) | 0.5 (0.8) |
| LVF_S_Persev, mean (SD) | 0.6 (1.0) | 0.5 (0.8) | 0.7 (1.3) |
| **Digit Span** |  |  |  |
| DigitSpan_max_span, mean (SD) | 6.1 (1.2) | 6.2 (1.5) | 5.9 (0.7) |
| DigitSpan_3correct, mean (SD) | 5.1 (1.0) | 5.1 (1.2) | 5.1 (0.9) |
| DualTask_digitspan_correct_single, mean (SD) | 12.4 (6.4) | 12.5 (6.0) | 12.2 (7.2) |
| DualTask_digitspan_incorrect_single, mean (SD) | 3.0 (2.8) | 2.8 (2.9) | 3.1 (2.7) |
| DualTask_digitspan_correct_double, mean (SD) | 12.4 (5.5) | 12.7 (5.6) | 12.1 (5.6) |
| DualTask_digitspan_incorrect_double, mean (SD) | 2.8 (2.8) | 2.5 (3.2) | 3.2 (2.2) |
| DualTask_boxes_single, mean (SD) | 135.8 (52.0) | 150.8 (44.4) | 116.3 (56.8) |
| DualTask_boxes_double, mean (SD) | 121.9 (51.5) | 137.2 (43.0) | 101.9 (56.8) |
| **Tower of Hanoi** |  |  |  |
| ToH_TotalItemsCorrect, mean (SD) | 8.0 (1.4) | 7.6 (1.7) | 8.5 (1.0) |
| ToH_TotalRawScore, mean (SD) | 19.7 (6.2) | 17.2 (5.7) | 23.0 (5.5) |
| ToH_ScaledScore, mean (SD) | 12.3 (4.2) | 10.5 (4.0) | 14.6 (3.5) |
| ToH_TimePerMoveRatio, mean (SD) | 3.5 (2.0) | 3.3 (1.4) | 3.7 (2.7) |
| ToH_TimePerMoveRatio_Scaled, mean (SD) | 8.9 (4.3) | 7.9 (4.4) | 10.3 (4.0) |
| ToH_MoveAccuracyRatio, mean (SD) | 1.3 (0.3) | 1.3 (0.4) | 1.2 (0.2) |
| ToH_MoveAccuracyRatio_Scaled, mean (SD) | 10.2 (3.3) | 9.2 (4.0) | 11.5 (1.5) |
| ToH_TotalRuleViolations, mean (SD) | 0.1 (0.5) | 0.2 (0.6) | 0.0 (0) |
| ToH_TotalRuleViolations_CPR, mean (SD) | 95.0 (16.8) | 91.1 (22.0) | 100.0 (0) |
| ToH_RuleViolationPerItemRatio_scaled, mean (SD) | 10.6 (1.7) | 10.3 (2.2) | 11.0 (0) |
| **Card Sorting Test** |  |  |  |
| Sort_WordsIncorrectlyRead, mean (SD) | 0.0 (0) | 0.0 (0) | 0.0 (0) |
| Sort_WordsNotUnderstood, mean (SD) | 0.0 (0) | 0.0 (0) | 0.0 (0) |
| Sort_TotalDescriptionScore, mean (SD) | 17.7 (5.5) | 18.8 (4.7) | 16.2 (6.4) |
| Sort_NumberCorrectConfirmedSorts, mean (SD) | 4.5 (1.4) | 4.8 (1.2) | 4.1 (1.7) |
| **Symbol Digit Modality Test** |  |  |  |
| SDMT_TotalCorrect, mean (SD) | 41.6 (17.6) | 44.6 (16.7) | 37.6 (18.8) |
| SDMT_SC_Error, mean (SD) | 0.3 (0.7) | 0.5 (0.9) | 0.1 (0.3) |
| SDMT_Incorrect, mean (SD) | 0.8 (1.7) | 0.8 (1.4) | 0.8 (2.2) |
